# Functional and mechanistic studies reveal MAGEA3 as a pro-survival factor in pancreatic cancer cells

**DOI:** 10.1186/s13046-019-1272-2

**Published:** 2019-07-08

**Authors:** Biswajit Das, Shantibhusan Senapati

**Affiliations:** 1 Tumor Microenvironment and Animal Models Lab, Institute of Life Sciences, Bhubaneswar, Odisha 751023 India; 2 Manipal Academy of Higher Education, Manipal, Karnataka India

**Keywords:** MAGEA3, Pancreatic cancer, CCL2, Survivin, Autophagy, Cancer testis antigen

## Abstract

**Background:**

In the era of personalized therapy, functional annotation of less frequent genetic aberrations will be instrumental in adapting effective therapeutic in clinic. Overexpression of Melanoma associated antigen A3 (MAGEA3) is reported in certain pancreatic cancer (PCA) patients. The major objective of the current study was to investigate the functional role of MAGEA3 in pancreatic cancer cells (PCCs) growth and survival.

**Methods:**

Using overexpression (tet-on regulated system and constitutive expression system) and knockdown (by siRNA and shRNA) approach, we dissected the mechanistic role of MAGEA3 in pancreatic cancer pathogenesis. We generated MAGEA3 expressing stable PCA cell lines and mouse primary pancreatic epithelial cells. MAGEA3 was also depleted in certain MAGEA3 positive PCCs by siRNA or shRNA. The stable cells were subjected to in vitro assays like proliferation and survival assays under growth factor deprivation or in the presence of cytotoxic drugs. The MAGEA3 overexpressing or depleted stable PCCs were evaluated in vivo using xenograft model to check the role of MAGEA3 in tumor progression. We also dissected the mechanism behind the MAGEA3 role in tumor progression using western blot analysis and CCL2 neutralization.

**Results:**

MAGEA3 overexpression in PCA cells did not alter the cell proliferation but protected the cells during growth factor deprivation and also in the presence of cytotoxic drugs. However, depletion of MAGEA3 in MAGEA3 positive cells resulted in reduced cell proliferation and increased apoptosis upon growth factor deprivation and also in response to cytotoxic drugs. The in vivo xenograft study revealed that overexpression of MAGEA3 promoted tumor growth however depleting the same hindered the tumor progression. Mechanistically, our in vitro and in vivo study revealed that MAGEA3 has tumor-promoting role by reducing macro-autophagy and overexpressing pro-survival molecules like CCL2 and survivin.

**Conclusion:**

Our data proves tumor-promoting role of MAGEA3 and provides the rationale to target MAGEA3 and/or its functional mediators like CCL2 for PCA, which may have a better impact in PCA therapy.

**Electronic supplementary material:**

The online version of this article (10.1186/s13046-019-1272-2) contains supplementary material, which is available to authorized users.

## Background

Pancreatic cancer (PCA) is of great concern for having six months of median survival period and five year survival of less than 5% [1]. In various cancers including PCA, the known common genetic alterations are still unable to completely explain the oncogenic process, which leads to redefining the genetic basis of the cancer cells [1–3]. The common genetic alterations involved in pancreatic cancer include activating mutation in *KRAS* and inactivating mutations in *TP53*, *CDKN2A* and *SMAD4*. In addition to the above common genetic changes, recently different set of genes are also reported to be associated with PCA pathogenesis [1, 2].

Expression of a group of germ cell genes, otherwise known as cancer-testis (CT) antigens are reported in different cancers. Their ectopic expression, in cancer cells is believed to be due to global demethylation during the course of cancer initiation and/or progression and also associated with the disease pathogenesis [3–7]. Identification of unique cancer-associated genes, which do not express in noncancerous somatic cells, may help to develop immunotherapy against pancreatic cancer [8]. Recent studies have also shown the functional significance of certain CT-antigens in different cancers [9, 10]. The less frequent but cancer-specific genes are now of great importance in designing personalized therapy [11, 12]. One of the most frequently reported germ cell genes across various cancer types (including PCA) is melanoma associated antigen A3 (MAGEA3) [13]. In the past, various studies have shed importance on the functional characterization of the cancer-specific gene MAGEA3; however, its functional role in PCA is yet to be elucidated [14–28].

Cancer cells possess the ability to reprogram their metabolism, which helps them to survive and proliferate under hostile condition [29]. During the transformation process, oncogenes help cancer cells to alter metabolism, and also make the cancer cells dependent on the altered metabolism provided by them (oncogenic dependency) [30, 31]. The oncogenes, involved in metabolic reprogramming in cancer are of great importance in developing targeted therapy [32, 33]. Multiple anti-cancer drugs targeting metabolism are in use and some are under trials [29]. Previous studies have shown the role of MAGEA3 in cancer cells metabolism [16, 17].

Overexpression of MAGEA3 has been reported in multiple malignancies including PCA [34–40]. Unlike malignancies such as melanoma, the frequency of MAGEA3 overexpression in PCA has been reported as moderate, which supports the known low immunogenic properties of PCA cells [41, 42]. In the recent past studies have shown potential pro-tumorigenic role of MAGEA3 in different other malignancies [15, 16, 23, 43, 44]. The poor prognosis of patients is also reported to be associated with MAGEA3 expression in different cancers including pancreatic cancer [5, 45, 46]. In the current study, we report the mechanistic role of MAGEA3 in pancreatic cancer cells (PCCs) growth and survival in the presence and absence of growth factor (GF). Moreover, the study provided experimental evidence that suggests the role of MAGEA3 in conferring chemo-resistance to PCCs.

## Materials and methods

### Cell culture

The cell lines used in this study are LNCap, BxPC3, AsPC1, MiaPaCa2, PANC1, SW1990, and HEK293T. Pancreatic cancer cell lines were procured through Sigma-Aldrich (ECACC authenticated cell lines) and were confirmed through STR profiling. Cells were routinely screened for mycoplasma contamination and were grown in a sterile humidified chamber with 5% CO_2_ and at 37 °C. LNCap and BxPC3 cells were maintained in RPMI media containing 10% FBS (Invitrogen), 100 units/mL penicillin and 100 mg/mL streptomycin; however AsPC1, MiaPaCa2, PANC1, SW1990 and HEK293T were grown in DMEM supplemented with above-mentioned concentration of FBS, penicillin and streptomycin.

Knockdown studies were conducted using siRNA transfection (Additional file 1: Table S2) and shRNA-stable cells. siRNA was transfected using RNAiMax (Additional file 1: Table S4) following manufacturers instruction. shRNA stable cells were generated using lentiviral particles of Lenti-sh-MAGEA3si1 or Lenti-sh-Contol.

### Isolation, characterization and genetic manipulation of mouse pancreatic epithelial cells

Untransformed cell lines like HEK293, NIH3T3 and immortalized primary epithelial cells are of instrumental in evaluating the function of a gene, especially in oncogenic context. In our study, we isolated and cultured mouse pancreatic epithelial cells using an established protocol [47]. Briefly, after harvesting the mouse pancreas in aseptic condition, the same was digested with collagenase type V enzyme solution (0.1 mg/mL) at 37 °C for 20 min. The enzymatic reaction was stopped; the cell pellet was washed and resuspended in culture media. The cells were seeded onto collagen-coated plates to favor the epithelial cells and grown in the presence of specific growth factors required for epithelial cells (Additional file 2: Figure S1a and S1b). The homogenous population of epithelial cells was obtained after third passage (Additional file 2: Figure S1c). The isolated cells, were confirmed for their epithelial origin by using E-cadherin as a marker (Additional file 2: Figure S1d) and manipulated with an expression construct of GFP or mouse KRAS^G12D^-HA or humanMAGEA3-HA or humanMAGEA3. The stable mouse primary pancreatic epithelial cells expressing above proteins (Additional file 2: Figure S1e and S1f) were established using puromycin (3 μg/mL) selection.

### Spheroid culture

Spheroids of control cells or MAGEA3 overexpressing stable cells were generated by hanging drop method in the presence or absence of doxycycline. After 72 h, the formed spheroids were transferred to 0.6% low melting agar-coated plates (to prevent attachment) and incubated at 37 °C, 5% CO_2_ with desired experimental conditions followed by further analysis.

### RNA isolation and cDNA synthesis

Total RNA was isolated from different cell lines after completing the appropriate duration of experiments using RNA easy kit following manufacturers instruction. DNA contamination was eliminated during RNA isolation by on-column DNA digestion using RNase-free-DNase set. Total RNA from different samples were quantified using UV-visible spectrophotometer (Eppendorf). Two microgram of RNA was used for cDNA synthesis using High capacity cDNA synthesis kit (Life Technologies).

### Quantitative PCR analysis

Gene expression analysis was done in LC-480 (Roche) platform. The used gene-specific primers (Additional file 1: Table S1) were procured from Euorofins Genomics.

### Generation of MAGEA3 overexpression construct

#### Non-Viral Constitutive expression system

The human (hu) MAGEA3 complete coding sequence (gi|16,877,053|gb|BC016803.1) was obtained from the NCBI database and primers with proper restriction sites were designed. To amplify *MAGEA3*, mRNA was isolated from LNCaP cells and cDNA was prepared. Then using the following primers the *MAGEA3* whole coding sequence was amplified.

**EcoRV**-huMA3F- 5′ GCG**GATATC**CATCATGCCTCTTGAGCAG and **XhoI**-huMA3-ST-R- 5′ GCG**CTCGAG**TCATCACTCTTCCCCCTCT or **XhoI**-huMA3-NST-R- 5′ GCG**CTCGAG**CTCTTCCCCCTCT).

The amplified product was gel-purified, ligated in pCR-Blunt II-TOPO (Amp^R^) vector and transformed. The positive clones were screened through colony PCR and restriction digestion and then sequenced before sub-cloning into the pCMV-3tag-3A expression vector. The gene was sub-cloned into pCMV-3tag-3A expression vector (Neo^R^) between the EcoRV and XhoI restriction enzyme sites. The gene was cloned into pCMV-3tag-3A vector with or without Flag-tag. The clones were screened through restriction digestion and positive clones were again sequenced to confirm the right orientation and ORF of the target gene.

#### Constitutive and Tetracycline regulated (Tet-On) lentiviral expression system

Human *MAGEA3* cds was cloned into pLenti-CMV-Puro-Dest vector (constitutive promoter) or pSIN-TRE-Lenti (TRE: tetracycline response element; tetracycline-regulated promoter) and muKRAS^G12D^ cds was cloned into pLenti-CMV-Puro-Dest vector (constitutive promoter) using gateway cloning strategy. Primers were designed along with the recombination sites (attB1 or attB2) flanking the gene-specific sequences. The primers used were listed (Additional file 1: Table S1). The PCR products attB1-*MAGEA3*-ST-attB2, attB1-*MAGEA3*-HA-attB2 and attB1-muKRAS^G12D^-HA-attB2 were amplified using Platinum *Pfx* proofreading enzyme PCR kit (Invitrogen); the PCR products were analyzed on 1% agarose gel, specific desired bands were excised and purified using gel purification kit (GE). 150 ng of purified PCR products were recombined to pDONR221 using BP clonase reaction mix (Invitrogen) at 25 °C for overnight. The reaction was stopped by incubating with proteinase K at 37 °C for 10 min. The products were transformed into *Stbl3* chemically competent *E.coli* cells and plated on agar plates containing 50 μg/mL of kanamycin. After overnight incubation at 37 °C, the single colonies on agar plates were picked and inoculated (LB broth media, 50 μg/mL kanamycin) for plasmid isolation. The isolated donor plasmids were quantified and sequenced. The donor plasmids containing attL1-*MAGEA3*-ST-attL2, attL1-*MAGEA3*-HA-attL2 or attL1- muKRAS^G12D^-HA-attL2 were recombined with the destination vector pLenti-CMV-Puor-Dest [48] or pSIN-TRE-Lenti (having attR1---attR2) using LR clonase reaction mix (Invitrogen). 150 ng of each plasmid was taken for recombination reaction and the reaction was set for overnight at 25 °C. After stopping the reaction with proteinase K as described above, the products were transformed into *Stbl3* competent cells, plated on agar plates containing 100 μg/mL of ampicillin and incubated at 37 °C for overnight. The single colonies were inoculated into LB broth (100 μg/mL, ampicillin) media and incubated at 37 °C, 200 rpm for overnight followed by plasmid isolation and sequencing. From the sequencing results, we confirmed the cloning of human *MAGEA3* cds with or without HA-tag into the pLenit-CMV-Puro-Dest constitutive lentiviral expression vector and tetracycline regulated expression vector pSIN-TRE-Lenti. The proper cloning of muKRAS^G12D^-HA into pLenit-CMV-Puro-Dest constitutive lentiviral expression vector was also confirmed through sequencing. The sequence of siRNA and shRNA used in this study are mentioned (Additional file 1: Table S2).

### Production of lentiviral particle of expression constructs

All lentiviral works were done in BSL2 facility after institutional biosafety committee approval (Institute of Life Sciences, Bhubaneswar). The generated tetracycline-regulated MAGEA3/MAGEA3-HA or constitutive MAGEA3/MAGEA3-HA/GFP/muKRAS^G12D^-HA expression constructs were packaged into lentiviral particles using packaging plasmids PMD2.G and pCMVR8.74. The detailed procedure for generation of lentiviral particles of the above said constructs is as follows. About 1.5 × 10^6^ number of HEK293T cells were seeded in 10 mL media (DMEM containing 10% FBS and 1% penicillin/streptomycin) into 10 cm tissue culture plate and incubated in a humidified incubator at 37 °C and 5% CO_2_. After 24 h of seeding, the media was changed; further, after 6 h, the cells were transfected with the expression constructs of MAGEA3 and packaging plasmids (PMD2.G and pCMVR8.74) using CalPhos™ Mammalian Transfection Kit (TaKaRa) as per the manufacturer’s instruction. Briefly for a single transfection reaction, 10 μg of target gene expression construct, 4 μg of PMD2.G and 6 μg of pCMVR8.74 were taken in an eppendorf tube, to which 293 μL of 0.1X TE buffer (pH -8) was added following the addition of 155 μL of dH_2_O into the cocktail mix. The above mix was vortexed and 50.2 μL of 2.5 M CaCl_2_ was added and again under vortexing condition 506 μL of 2xHBSS was added dropwise. The cocktail mix was vortexed well and kept at RT undisturbed for 5 min. After 5 min the mixture was added to the HEK293T cells dropwise and the cells were incubated for 18 h at 37 °C in a humidified incubator supplied with 5% CO_2_. After 18 h, 10 mL of fresh complete media was added to the transfected cells and incubated at 37 °C in a humidified incubator supplied with 5% CO_2_ for 24 h. After 24 h the culture media was collected and filtered through 0.45 μm syringe filter. 3 mL (1/3rd of the filtrate) of Lenti-X concentrator (TaKaRa) was added to the filtrate, mixed by gentle inversion and incubated overnight at 4 °C, followed by centrifugation at 1500 x g for 45 min at 4 °C to obtain an off white pellet. The pellet was reconstituted in 200 μL of incomplete DMEM media, aliquoted into 20 μL in eppendorf tubes and stored at − 80 °C till use.

### Proliferation assay and cell viability assay

To study the growth pattern/proliferation rate of the cancer cells MTT assay was done. For MTT assay cells were seeded at a density of 800 cells/well in 96 well tissue culture plates, grown for indicated days in presence or absence of doxycycline. MTT was added every day to three wells of each condition and incubated at 37 °C for 4 h, the crystal was dissolved in DMSO and the absorbance was taken at 570 nm. The graph represents the growth rate.

We did MTT assay to determine the percentage of cells survived in serum-free condition (0% FBS) as compared to growth factor rich condition (10% FBS) in presence or absence of MAGEA3 in pancreatic cancer cells. Two thousand cells per well of 96 well plate, were seeded in 100 μL of complete media, and after attachment 100 μL of complete media containing 200 ng/mL of doxycycline/tetracycline was added to each well to make a final concentration of 100 ng/mL doxycycline in complete media and incubated for 4 h in humidified incubator at 37 °C containing 5% CO_2_, which led to the expression of MAGEA3-HA/MAGEA3 in the generated stable cell lines (AsPC1-TRE-MAGEA3-HA/ AsPC1-TRE-MAGEA3) prior to subjecting them into serum starvation. After 4 h of doxycycline treatment in 10% FBS containing complete media MTT was added and incubated for 4 h to get the A_570nm_ for the zero day i.e. before starvation; at the same time, 10% FBS containing media was replaced with 0% FBS media with or without doxycycline. MTT reading was taken at every 24 h of interval in four different conditions i.e. 10% FBS-Dox^−^, 10% FBS-Dox^+^, 0% FBS-Dox^−^ and 0% FBS-Dox^+^ for each cell type (AsPC1-TRE-MAGEA3-HA/ AsPC1-TRE-MAGEA3/AsPC1/ AsPC1-GFP). Similarly, the cell viability assay was carried out for MiaPaCa2 and PANC1 cells. The percentage of survival in 0% FBS condition (Fig. 2 and Additional file 4: Figure S3) is calculated as follows:

$$ \%\mathrm{survival}\ \mathrm{in}\ 0\%\mathrm{FBS}{\mathrm{Dox}}^{\hbox{-} /+}=\left({\mathrm{A}}_{570\mathrm{nm}}0\%\mathrm{FBS}\ {\mathrm{Dox}}^{\hbox{-} /+}/{\mathrm{A}}_{570\mathrm{nm}}10\%\mathrm{FBS}\ {\mathrm{Dox}}^{\hbox{-}}\right)\times 100 $$

In case of spheroids, the number of viable cells present per spheroid was determined by dissociating the spheroids followed by counting on haemocytometer after trypan blue staining.

### Crystal violet assay

For crystal violet assay, 50 thousand cells were seeded per well of 6 well plate or 35 mm culture dish. After the appropriate experimental duration, cells were stained with 0.5% crystal violet solution, dried, imaged; crystal violet was dissolved in 10% acetic acid and quantified by taking absorbance at 540 nm.

### Apoptosis assay

Annexin-V and PI were used to detect apoptotic cell death. Cells, after the appropriate duration of treatment or condition were collected and washed with PBS. The cells were stained with annexin-V-FITC only, PI only or annexin-V-FITC + PI solution and analyzed in flowcytometer (BD FACS Calibur).

### Immunoblotting

Protein lysate was prepared in RIPA buffer (Radio-Immuno-Precipitation Assay buffer; 20 mM Tris-HCl (pH 7.5), 150 mM NaCl, 1 mM Na_2_EDTA, 1 mM EGTA, 1% NP-40, 1% sodium deoxycholate, 2.5 mM sodium pyrophosphate, 1 mM beta-glycerophosphate, 1 mM Na_3_VO_4_, 1 μg/ml leupeptin) containing protease inhibitor and phosphatase inhibitor and stored at − 80 °C until use. Proteins were first separated in SDS-polyacrylamide gel and then transferred to 0.45 μm PVDF membrane. The blots were incubated with desired primary antibodies (Additional file 1: Table S3) at 4 °C for overnight in gentle shaking followed by one hour incubation with peroxidise-conjugated secondary antibody at room temperature and then the bands were detected in chemidoc or X-ray film using ECL substrate.

### Immunofluorescence staining

Cells were seeded on poly-L-lysine coated coverslips placed in 35 mm tissue culture plates and after the end of the experimental condition, the cells were washed with PBS followed by fixation and permeabilization using ice-cold methanol:acetone (1:1) at − 20 °C for 10 min. For E-cadherin, the cells were fixed with 4% formalin but not permeabilized. Then, after washing with PBS the cells were blocked with 2% BSA (in PBS) for 30 min at room temperature with gentle shaking. After blocking, the cells were incubated with primary antibody at 4 °C overnight. Then the coverslips were washed with PBS and incubated with secondary antibody conjugated with alexafluor 594 (red) or alexafluor 488 at RT for 45 min in dark. Finally, the coverslips were washed two times with PBS and incubated with Phalloidin-555 to stain the cytoskeleton and/or mounted with ProLong® Gold Antifade Mountant with DAPI (nuclear stain). The slides were visualized under fluorescence microscope (ApoTome.2, ZEISS) and images were captured.

### Xenograft mouse model

The animal experiment reported in this study was approved from the institutional animal ethical committee (IAEC), Institute of Life Sciences, Bhubaneswar, India. The female nude mice were purchased from Vivo Bio Tech Ltd., Telangana, India and maintained in animal house facility at ILS. Two million cancer cells were injected subcutaneously on the rear flanks and grouped as indicated below. AsPC1-GFP D−/AsPC1-TRE-MAGEA3-HA D- (Left flank/Right flank), *n* = 5; AsPC1-GFP D+/AsPC1-TRE-MAGEA3-HA D+ (Left flank/Right flank), received 50 ng/mL doxycycline in drinking water, n = 5; AsPC1-pLentiMAGEA3-HA/AsPC1-pLentiMAGEA3 (Left flank/Right flank), *n* = 5. BxPC3-GFP/ BxPC3-MAGEA3 (Left flank/Right flank), n = 5; BxPC3-sh-ve/BxPC3-sh1 (Left flank/Right flank), *n* = 3. Tumor volume and body weight were recorded on every alternate day. Tumor volume was calculated using the formulae Volume (mm^3^) = (Length x Width^2^)/2. Part of each tumor was snap frozen and remaining were fixed in 10% buffered formalin solution for histological analysis. BrdU was injected into the peritoneum at 2 mg/20 g mice (100 mg/kg of body weight) one hour prior to the sacrifice of animals.

### Tissue or spheroid processing and immunohistochemistry

After the end of experimental condition time point, spheroids were fixed in 10% buffered formalin at 4 °C for 48 h and then processed and embedded in paraffin and block was made. Sections of 4 μm thickness were made and processed for immunohistochemical staining. The tumors isolated from animals were kept in formalin for 7 days and then processed for embedding into paraffin blocks followed by sectioning and immuunohistochemical staining procedures as reported earlier [49]. For BrdU staining, the tissue sections were deparaffinized, rehydrated followed by washing with PBST (PBS + 0.1% TritonX-100). After washing the sections were treated with proteinase K (20 μg/mL in PBS) followed by washing with PBS. Then the sections were incubated with 2 N HCl and then with sodium borate solution (0.1 M). Further, the sections were washed with PBS and treated with 3% H_2_O_2_. Again, the sections were washed with PBS and blocked with the mouse on mouse blocking solution. Then, the primary antibody against BrdU was applied and incubated overnight at 4 °C. Afterward, the sections were washed with PBS and incubated with biotinylated secondary antibody and processed with DAB based immunohistochemical detection methodology, which includes incubation with ABC reagent and developed with DAB. After staining and mounting with cover-slip, the slides were visualized under a microscope (Leica ICC500) and images were captured. The number of cells positive for staining were quantified using ImageJ software.

### CCL2 ELISA

An equal number (2 × 10^5^) of control and MAGEA3 stable cells were seeded onto 6 well culture plates and cultured with equal volume (3 mL) of culture media. After completion of the required experimental time period, the culture media (conditioned media, CM) were collected and assayed for the presence of human CCL2 using sandwich ELISA (R&D systems) following manufacturers instruction.

### Statistics

All the in vitro experiments were performed thrice and the data presented here were from a representative experiment as the mean ± standard error of the mean (SEM). The data were analysed statistically by Student’s *t*-test or two-way ANOVA using GraphPad prism 5.00 (GraphPad Software, Inc., San Diego, USA).

## Results

### MAGEA3 expression in different parental pancreatic cancer cell lines and their derivatives

It has been reported that MAGEA3 is expressed in a sporadic pattern in tumor tissues, which limits the strategy of immunotherapy using MAGEA3 as a candidate [46, 50, 51]. We sought to check the expression status of MAGEA3 in different PCA cell lines. We observed almost undetectable level of MAGEA3 in AsPC1, MiaPaCa2, SW1990 and PANC1 pancreatic cancer cells both at *mRNA* (Additional file 3: Figure S2a) and protein level (Additional file 3: Figure S2b). However, we observed a high level of MAGEA3 in BxPC3 pancreatic cancer cells (Additional file 3: Figure S2a and S2b). The data shows cell line dependent expression pattern of MAGEA3 in PCA, and further supports the known sporadic nature of its expression in different other cancers.

To investigate the functional role of MAGEA3 in PCA, we adopted ectopic gene expression and silencing approaches. Previously, constitutive overexpression of MAGEA3 in different cancer cell lines has been used to investigate its functional role. Experimentally, constitutive or regulated overexpression of genes has their intrinsic strength and weakness. Hence, to have a convincing conclusion about the function of MAGEA3 in PCCs, we generated MAGEA3 overexpressing cell lines that express MAGEA3 in a constitutive or regulated fashion. Further to distinguish the ectopically expressed MAGEA3 from endogenous MAGEA3, we incorporated HA-tag at the C-terminal end of the MAGEA3 open reading frame. The stable cell line with inducible MAGEA3 construct (cell line name-TRE-MAGEA3/MAGEA3-HA) expressed MAGEA3 at basal level (Additional file 3: Figure S2c-f) and the level increased in response to doxycycline with a minimum dose of 50 ng/mL (Additional file 3: Figure S2c). All the experiments were carried out with 100 ng/mL of doxycycline for optimal MAGEA3 expression wherever required. Doxycycline of 100 ng/mL induced the MAGEA3 expression within 4 h of treatment and continues to induce the protein expression at least till 10 days if treated once (Additional file 3: Figure S2d). In generated stable cells, MAGEA3 was expressed in native form (Additional file 3: Figure S2e and S2g) or with a HA-tag at its C-terminal (Additional file 3: Figure S2c, S2d and S2g) and importantly both the forms were detected by anti-MAGEA3 antibody with a little shift of HA-tag form (Additional file 3: Figure S2g). Thus, we confirmed that the generated constructs effectively express MAGEA3 and is detectable through anti-MAGEA3 antibody. At the same time, to rule out the non-specific side effect of overexpression system and confirm the findings of the overexpression studies, we used siRNA/shRNA mediated knockdown of MAGEA3 in BxPC3 cells. We tried two siRNA sequences (Additional file 1: Table S2) targeting MAGEA3 [16] and both the siRNA were equally efficient to reduce *MAGEA3* expression in BxPC3 pancreatic cancer cells (Additional file 3: Figure S2h and S2i).

### Ectopic MAGEA3 expression does not affect the pancreatic cancer cells’ growth but knockdown affects the viability of pancreatic cancer cells in vitro

**Fig. 1:**
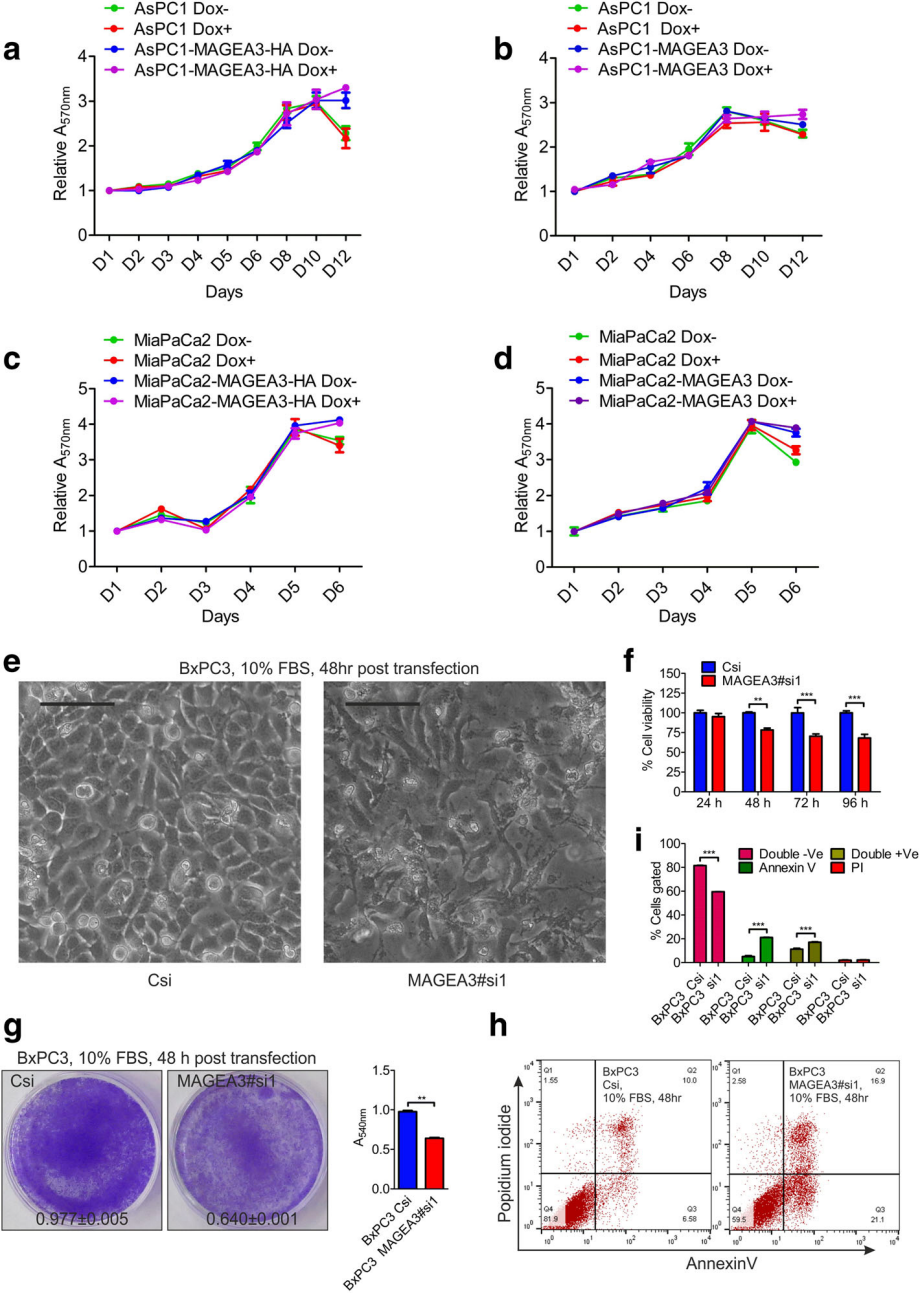
Under normal culture condition, ectopic expression of MAGEA3 has no effect on the pancreatic cancer cells’ proliferation but knockdown affects the viability in vitro*.* **a**-**d** MTT assay showing the rate of proliferation of the indicated pancreatic cancer cells, Y-axis showing the absorbance relative to Day 1 (D1); X-axis showing the indicated days. **e** Representative bright field micrograph showing change in morphology upon MAGEA3 depletion in BxPC3 cells, scale bar = 100 μm. **f**-**i** MTT assay showing the decrease in viable cells upon MAGEA3 knockdown (**f**), crystal violet stained plate image and the corresponding bar graph showing reduction in cell number upon MAGEA3 depletion (**g**; mean ± SEM, *n* = 3). AnnexinV and propidium iodide (FACS analysis) assay showing increased number of apoptotic and pre apoptotic cells upon MAGEA3 depletion (**h** and **i**). ** = *p* < 0.005, *** = *p* < 0.001, *n* = 3

The tumor-promoting role of MAGEA3 was assessed firstly by in vitro cell proliferation assay in complete growth medium (10% FBS). We observed that ectopic expression of MAGEA3 in MAGEA3 non-expressing PCCs (AsPC1, MiaPaCa2) did not alter the rate of proliferation in nutrient-enriched condition (Fig. 1a-d). However, when we knocked-down the said molecule in BxPC3 PCC, we observed a reduction in cell viability (Fig. 1e-i), which indicates the dependency of *MAGEA3* expressing cells (i.e. BxPC3) on MAGEA3 for their survival. This cell-line depended role of MAGEA3 in PCCs cultured in GF-enriched conditions indicates that presence and/or absence of other genetic aberrations might influence MAGEA3 function in these cells.

### MAGEA3 provides survival advantage to pancreatic cancer cells in growth factor deprived condition

**Fig. 2:**
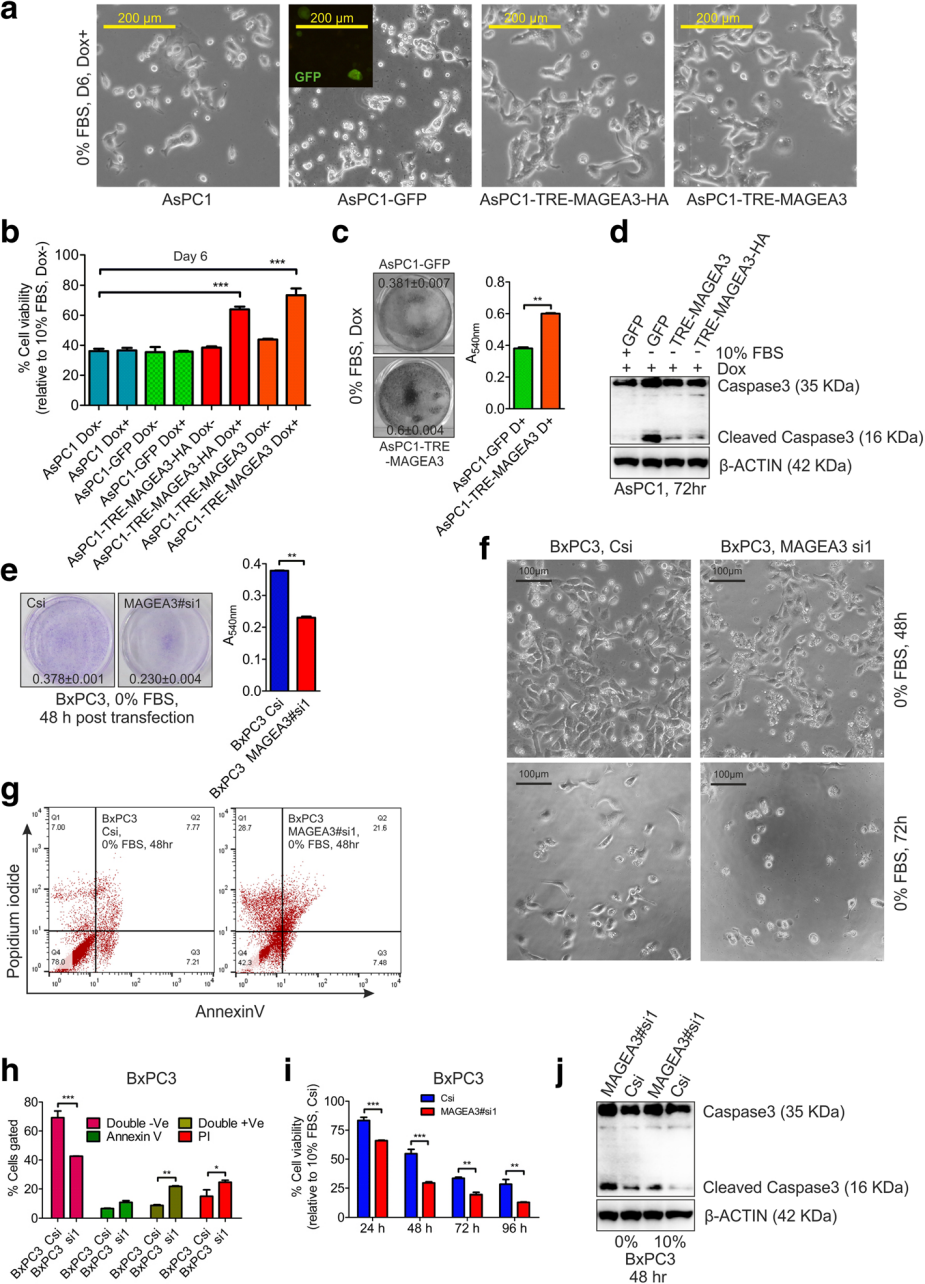
MAGEA3 provides survival advantage to growth factor deprived pancreatic cancer cells in 2D cell culture model. **a** Bright field images of serum starved pancreatic cancer cells showing more number of cells when MAGEA3 is expressed ectopically. “Dox+” refers to doxycycline treatment (100 ng/mL). Scale bar = 200 μm. **b** MTT assay showing the fraction of viable cells at indicated condition. “Dox+” refers to doxycycline treatment (100 ng/mL). *** = *p* < 0.001, *n* = 3. **c** Representative crystal violet stained images for the indicated condition for 72 h. Dox refers to doxycycline treatment (100 ng/mL). The values represents Mean ± SEM relative to 10% FBS + Dox, *n* = 3. **d** Immunoblot showing level of apoptosis in ectopically expressing MAGEA3 and control cells in response to growth factor deprivation. **e**, **f** Representative crystal violet stained plate image along with the bar graph (e; Mean ± SEM, *n* = 3) and bright field image (scale bar = 100 μm) (f) for the indicated condition.**g**-**i** FACS analysis for AnnexinV and propidium iodide stained cells (g and h) and MTT assay (i) showing more cell death upon MAGEA3 depletion, * = *p* < 0.05, ** = *p* < 0.005, *** = *p* < 0.001, *n* = 3. **j** Immunoblot showing level of apoptosis in MAGEA3 depleted BxPC3 cells

Excessive extracellular matrix deposition, otherwise known as desmoplasia in tumor tissues is a hallmark of PCA [52]. The desmoplasia in PCA tissues reduces nutrient diffusion and the cells residing towards the core of the tumor or away from blood vessels encounter metabolic stress due to nutrient and GF-deprivation [53]. However, the cells devised with gene signatures associated with metabolic pathways or cancer stem cell markers may have a survival advantage in such condition. So, we thought to investigate the role of MAGEA3 in pancreatic cancer cells under GF-deprived condition using both monolayer (2D) culture model and spheroid (3D) model. In the 2D culture model, the GF-deprivation condition is created by simple serum withdrawal from the culture media. In the 2D culture model, we found more fractions of viable cells after GF-deprivation in ectopically expressed MAGEA3-stable cells in comparison to parental/control cells (Fig. 2a-c and Additional file 4: Figure S3a, S3b and S3d). Importantly, when we knocked-down MAGEA3 in BxPC3 cells and subjected to GF-deprivation, the viability is greatly reduced (Fig. 2e-i). Further, we observed a decrease in apoptotic marker cleaved caspase3 in ectopically expressing MAGEA3 stable cells (Fig. 2d), but the same cleaved caspase3 was found to be increased upon MAGEA3 depletion (Fig. 2j). In the spheroid model, the gradient decrease of nutrient and GF-deprivation is created in vitro to mimic a tumor model having a hyper-proliferative peripheral zone. In spheroid culture model we observed bigger size of the spheroids of the ectopically expressing MAGEA3 stable cells in comparison to parental or stable GFP expressing control cells (Additional file 5: Figure S4a). Further, the trypan blue assay showing more viable cells in spheroids of MAGEA3 stable cells (Additional file 5: Figure S4b and S4c), corroborates with the findings of the aforementioned 2D culture studies. In spheroid model, apart from the proliferating peripheral zone we observed more number of Ki67 positive cells towards the core zone in MAGEA3 expressing stable cells in comparison to stable GFP expressing cells (Additional file 5: Figure S4d and S4e) which leads to overall increase in the viable proliferative (Ki67 positive) cells in spheroids of MAGEA3 overexpressing stable cells.

### MAGEA3 inhibits autophagy in growth factor deprived pancreatic cancer cells

**Fig. 3:**
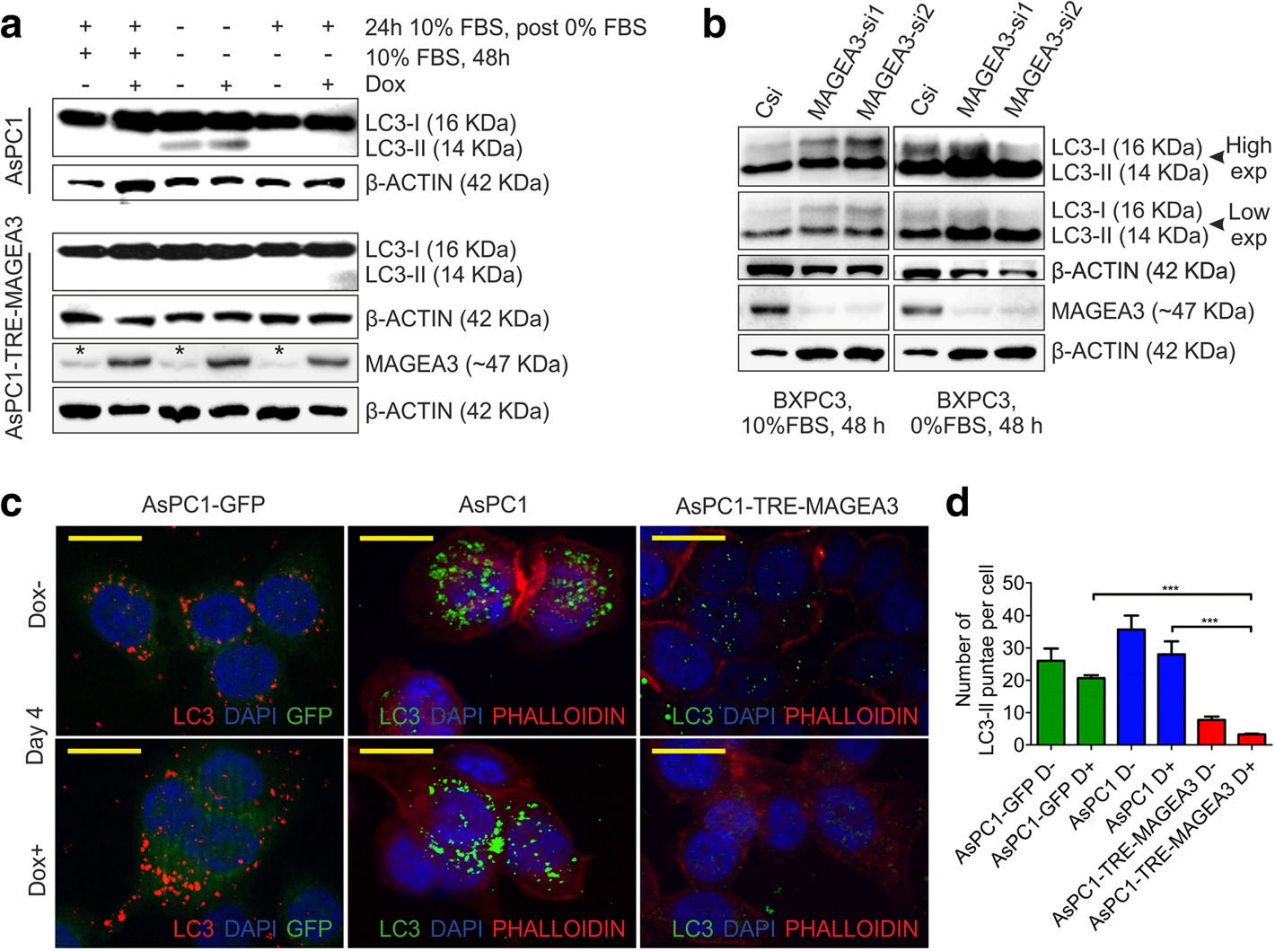
MAGEA3 inhibits autophagy in growth factor deprived pancreatic cancer cells. **a**-**b** Immunoblot showing decreased autophagic marker LC3-II in cells ectopically expressed with MAGEA3 (**a**) and the same LC3-II is increased upon MAGEA3 knockdown in BxPC3 cells (**b**). Star above the bands indicates the leaky expression of MAGEA3. **c, d** Immunofluorescence staining showing endogenous LC3-II puntae (**c**, scale bar = 25 μm) and the bar graph showing the corresponding quantification using ImageJ software (**d**); *** = *p* < 0.001, *n* = 10

It is well established that growth factor and/or nutrient deprivation leads to autophagy in different cells [54] and autophagy helps in clearing the stress granules with miss-folded proteins or protein aggregates from the cells in oxidative stress [55]. Autophagy, in one hand, helps the cells in clearing the unwanted components and recycles the bio-molecules; in another hand during growth factor and/or nutrient deprivation may lead to cell death due to excessive autophagy (macroautophagy) [56, 57]. Thus we wanted to check the role of MAGEA3 in autophagy hypothesizing that MAGEA3 expression in PCCs might regulate the autophagy process in these cells and thus provide a survival advantage. To address this hypothesis, first we checked the protein level of autophagy marker LC3-II in the PCCs subjected to GF-deprivation and we observed that LC3-II level is more in parental or GFP expressing control cells in comparison to MAGEA3 overexpressing cells (Fig. 3a, c, d and Additional file 4: Figure S3c, S3e and S3f). Further, in contrast to MAGEA3 expressing stable cells, the LC3-II level is also up-regulated in parental/GFP cells upon rapamycin treatment in combination with serum withdrawal (Additional file 6: Figure S5e). Upon MAGEA3 knockdown in BxPC3 cells, LC3-II is observed to be up-regulated in the presence or absence of growth factor (Fig. 3b). Together, the data suggest that MAGEA3 blocks autophagy in PCCs. However, sustained blockage of autophagy during GF-deprivation may be lethal to the cells due to excessive deposition of stress granules [56, 57]. We analyzed the fraction of viable cells of MAGEA3 overexpressing or depleted cells in response to autophagy inducers (rapamycin and torin2) or autophagy inhibitors (bafilomycinA1 and wortmannin). The results presented showed that MAGEA3 overexpressing cells has less autophagy (Additional file 6: Figure S5e) and more viable cells (Additional file 6: Figure S5a and S5b) in response to autophagy inducers, however inhibiting autophagy using autophagy inhibitors did not altered the fraction of viable cells in both AsPC1 control as well as MAGEA3 overexpressing stable cells. When we depleted MAGEA3 in BxPC3 cells we observed a decrease in viable cells upon torin2 treatment. Although bafilomycinA1 was able to rescue the cells from autophagic cell death upon MAGEA3 depletion, but wortmannin fails to do so at the used concentration (Additional file 6: Figure S5c and S5d). We observed more autophagic flux in case of parental cells in comparison to cells that express MAGEA3 ectopically (Additional file 6: Figure S5e). The above results showed that inhibition of autophagy was beneficial for cell survival during GF-deprivation and further we hypothesize that MAGEA3 expressing cells may have minimised the autophagic catabolic process and activated some signalling pathway to survive during prolonged GF-deprivation.

### MAGEA3 activates alternative survival pathway in growth factor deprived condition

**Fig. 4:**
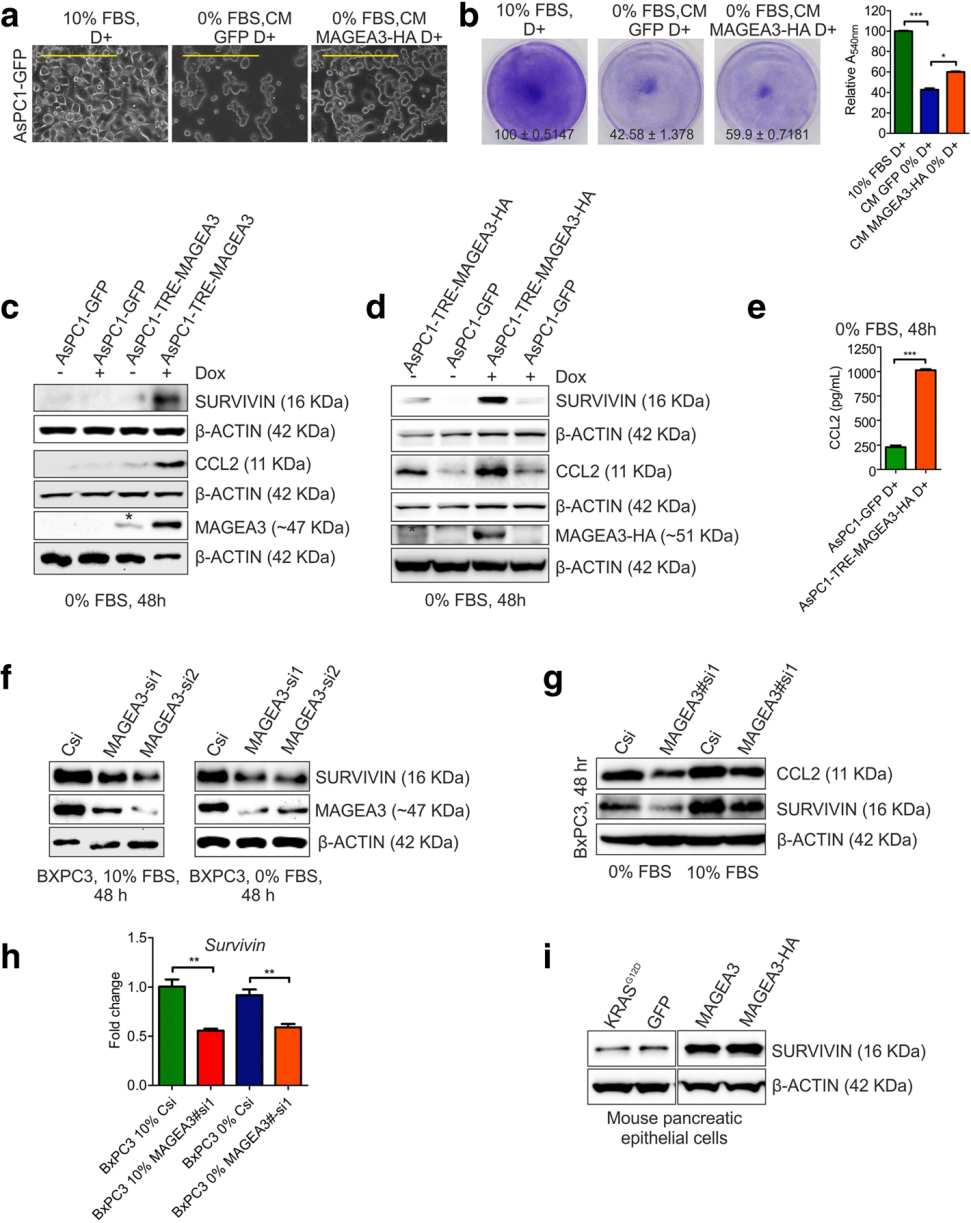
MAGEA3 is involved in cell survival pathway that helps the cancer cells to survive during growth factor deprivation. **a**, **b** Bright field micrographs (**a**, scale bar 200 μm, 10X objective) of AsPC1-GFP i.e. control cells and crystal violet stained plate images (**b**, Mean ± SEM, *n* = 3) showing the survival advantage provided by the conditioned media from AsPC1-MAGEA3-HA cells (72 h, fresh conditioned media after 24 h). CM, Conditioned media. The bar graph represents the quantification of the crystal violet assay. The percentage is calculated relative to the 10% FBS + doxycycline condition (The value is Mean ± SEM, *n* = 3). **c**, **d** Immunoblot showing up-regualtion of CCL2 and survivin upon ectopic expression of MAGEA3. Star above the band indicates the leaky expression of MAGEA3 or MAGEA3-HA protein. **e** The bar graph represents the concentration of human CCL2 present in the conditioned media of control or MAGEA3-HA overexpressing cells. D+ represents doxycycline treatment (100 ng/mL). *** = *p* < 0.001, *n* = 3. **f**-**h** Immunoblot (**f** and **g**) and qPCR (**h**) analysis confirmed the down regulation of survivin and CCL2 in BxPC3 cells upon MAGEA3 knockdown. **i** Immunoblot shows increased expression of survivin upon MAGEA3 expression in mouse pancreatic epithelial cells

**Fig. 5:**
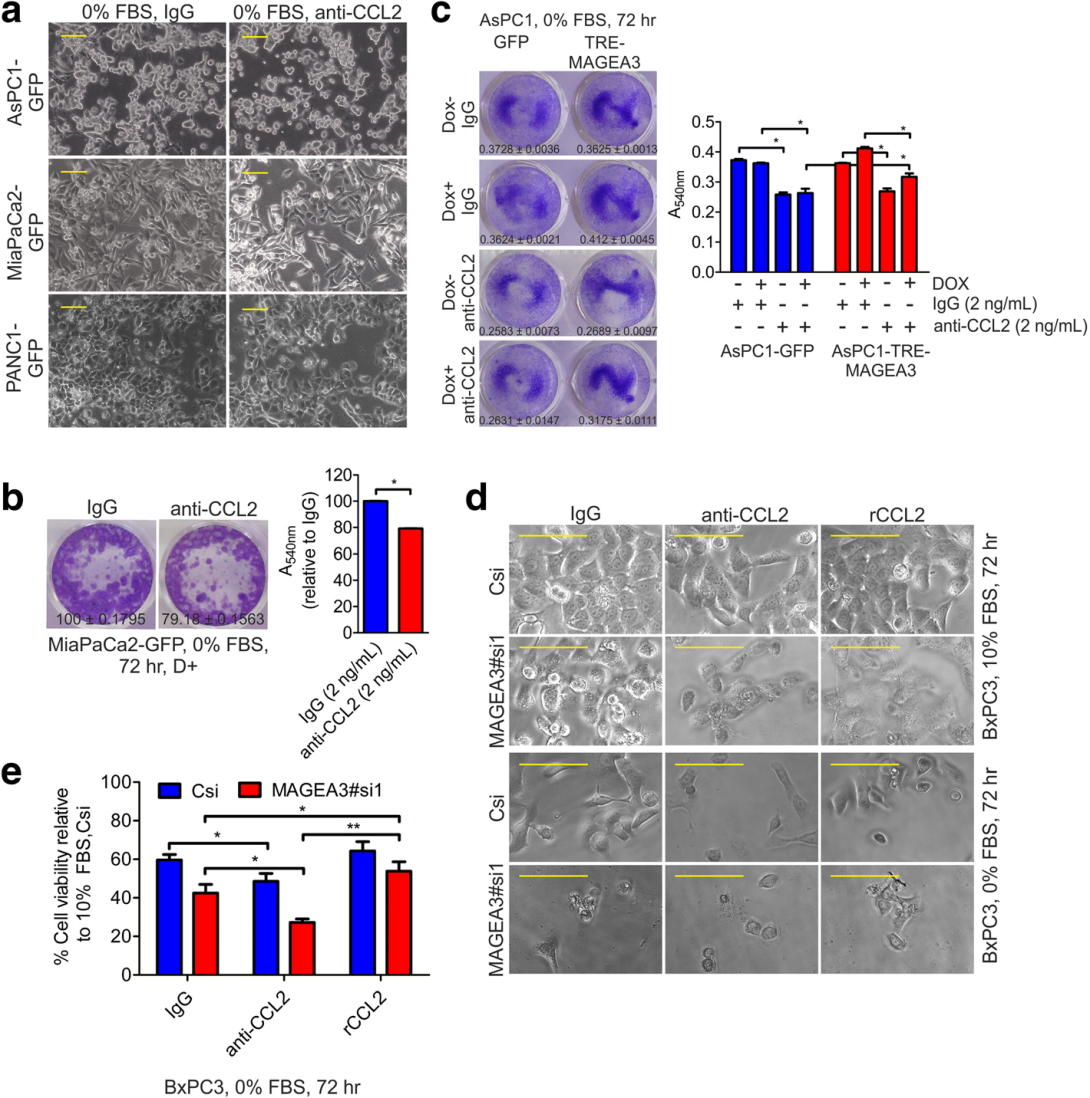
CCL2 neutralization reduced the cell viability in MAGEA3 non-expressing or MAGEA3-depleted cells. **a** Bright field images (scale bar = 100 μm, 20X objective) of pancreatic cancer cells shows decreased number of cells upon treatment with of CCL2 neutralizing antibody (2 ng/mL, every 24 h, 72 h treatment) in the absence of growth factors (0% FBS). **b**, **c** Crystal violet stained plate images showing the decreased number of cells upon CCL2 neutralization (anti-CCL2 antibody or IgG, 2 ng/mL, every 24 h, 72 h treatment) in both control and MAGEA3-expressing cells. In bar graph, the values represents Mean ± SEM,* = *p* < 0.05, *n* = 3. **d**, **e** Bright field images (**d**, scale bar 100 μm, 20X objective) and MTT assay (**e**) collectively shows the decrease in cell viability in response to CCL2 neutralization (anti-CCL2 antibody or IgG, 2 ng/mL, every 24 h, 72 h treatment) alone and further decreased upon MAGEA3 knockdown which can be reversed by supplementing with recombinant CCL2 (rCCL2, 1 ng/mL)

MAGEA3-TRIM28 mediated proteasomal degradation of proteins like AMPKα1 or FBP1 are known mechanisms through which MAGEA3 provides a survival advantage to different cancer cells [16, 20]. At the same time, studies have already demonstrated the involvement of various secretory factors produced by cancer cells in conferring survival advantage under GF-deprivation. Hence, we were curious to explore the possibility of any secretory factors involved in the aforementioned MAGEA3 mediated survival advantage to GF-deprived pancreatic cancer cells. To check the hypothesis, conditioned medium (CM) harvested from the GF-deprived MAGEA3 overexpressing AsPC1 cells or AsP1-GFP (MAGEA3 non-expressing) control cells was added to the AsPC1-GFP control cells under GF-deprivation. The results obtained from this study showed that CM obtained from the MAGEA3 overexpressing cells conferred a significant survival advantage to the AsPC1-GFP GF-deprived control cells (Fig. 4a and b). Previous studies have shown that CCL2 secreted by prostate cancer cells confer a survival advantage to these cells [58, 59]. Under GF-deprivation CCL2 is also known to suppress unwanted excessive autophagy and upregulates expression of proteins like survivin that confer a survival advantage to cancer cells [59–61]. PCCs are known to express CCL2 and its receptor CCR2 [62]. Hence, we got curious to check, if MAGEA3 expression has any effect on CCL2 expression in PCCs or not? The data (Fig. 4c-d and g) clearly demonstrated that MAGEA3 positively regulated CCL2 expression in PCCs. Moreover, MAGEA3-mediated upregulation of CCL2 expression also matched with MAGEA3-mediated survivin expression in these cells including mouse primary pancreatic epithelial cells (Fig. 4c-d and f-i). We also checked the level of human CCL2 in the conditioned media and found increased level of soluble human CCL2 upon MAGEA3 overexpression in GF-deprived condition (Fig. 4e). Further, to investigate the functional role of CCL2 in the survival of PCCs under GF deprivation AsPC1, MiaPaCa2, PANC1 and BxPC3 cells were subjected to complete GF deprivation in presence and absence of CCL2 neutralizing antibody or rCCL2 protein. After, 72 h of GF-derivation the cells were visualized through bright field microscopy (Fig. 5a and d) and the viability of the cells were checked by crystal violet assay (Fig. 5b and c), and MTT assay (Fig. 5e). The results clearly showed that CCL2 indeed plays a significant role in the survival of pancreatic cells in complete GF-deprived conditions, thus the viability is decreased upon CCL2 neutralization using CCL2 neutralizing antibody. Together, these data suggest that MAGEA3-mediated upregulation of CCL2 in PCCs might have a critical role in the survival of PCCs.

### Role of MAGEA3 in tumor progression in vivo

**Fig. 6:**
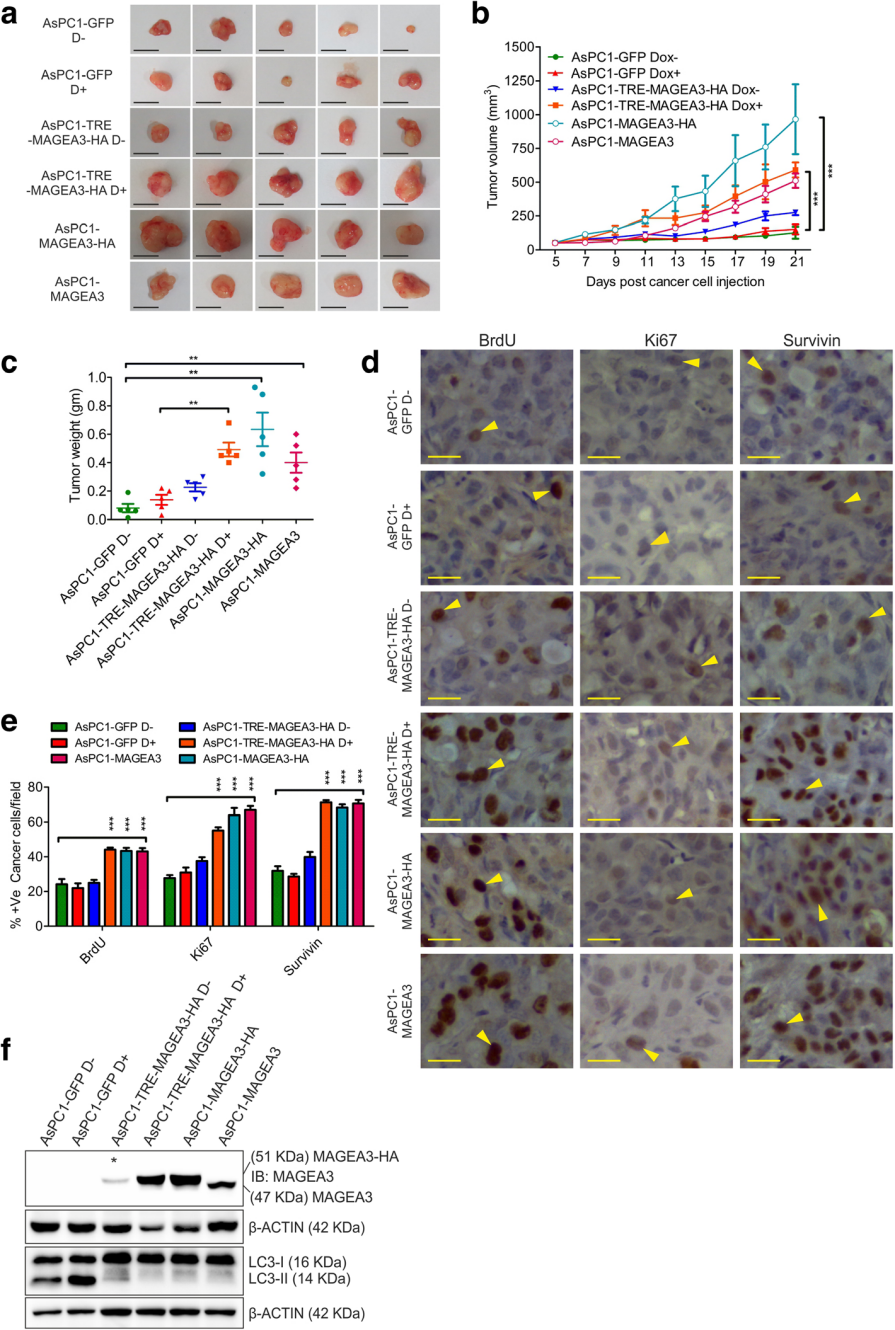
MAGEA3 expression results into increased tumor progression in vivo*.* **a** Digital images of the whole tumor isolated from mice; scale bar =1 cm, AsPC1-MAGEA3 or AsPC1-MAGEA3-HA refers to AsPC1-pLentiMAGEA3 or AsPC1-pLentiMAGEA3-HA in constitutive expression system. **b** The graph showing tumor growth kinetics in vivo. *** = *p* < 0.001, *n* = 5. **c** The graph showing the tumor weight measured after isolation. ** = *p* < 0.005, *n* = 5. **d, e** Immunohistochemical analysis showing representative images of BrdU, Ki67 and survivin positive cells in the tumor tissues of different groups (**d**). Quantification of cells positive for BrdU, Ki67 and survivin are presented through bar graphs (**e**). Scale bar = 25 μm. *** = *p* < 0.001, *n* = 5. **f** Immunoblot of protein samples from tumor tissue showing reduced authophagic marker LC3-II in MAGEA3 expressing xenografts. Star above the band indicates the leaky expression of MAGEA3-HA protein

To check the pro-survival role of MAGEA3 in PCCs in vivo*,* we used xenograft model. Corroborating with the in vitro results, PCCs with high MAGEA3 expression grew faster than control cells (Fig. 6a-c). Estimation of tumor weights showed a significantly higher tumor weight of MAGEA3 overexpressing tumors than controls. Moreover, quantification of BrdU-incorporation and Ki67 expression in PCCs showed that MAGEA3 overexpressing tumors had more viable and proliferating cells than control tumors (Fig. 6d and e). Estimation of survivin expression also showed a significantly higher level of survivin in MAGEA3 overexpressing tumors (Fig. 6d and e). In this study, the inclusion of AsPC1-GFP cells with or without Dox treatment helped us to confirm that the observed pro-tumorigenic effect upon MAGEA3 overexpression was not a consequence of ectopic expression of an exogenous protein, and the used dose of doxycycline (Dox) itself had no significant effect on AsPC1 cells growth in vivo. The autophagy level also found to be reduced upon MAGEA3 overexpression (Fig. 6f) which corroborated with the in vitro data. The tumor promoting role of MAGEA3 was further examined in MAGEA3 positive cell line. Interestingly, overexpression of MAGEA3 in MAGEA3 positive cell line BxPC3 further enhanced the tumor progression in vivo (Additional file 7: Figure S6a-S6c). However, the tumor progression was hindered when MAGEA3 was knocked down in BxPC3 cell line (Additional file 7: Figure S6d-S6f). The immunohistochemical analysis of tumor samples from BxPC3 xenografts showed enhanced BrdU and survivin positive cells upon MAGEA3 overexpression but was lowered in MAGEA3 depleted xenografts (BxPC3-sh1) compared to control tumors (Additional file 7: Figure S6i and S6j).

### Targeting MAGEA3 enhanced cell death to therapeutics in vitro

**Fig. 7:**
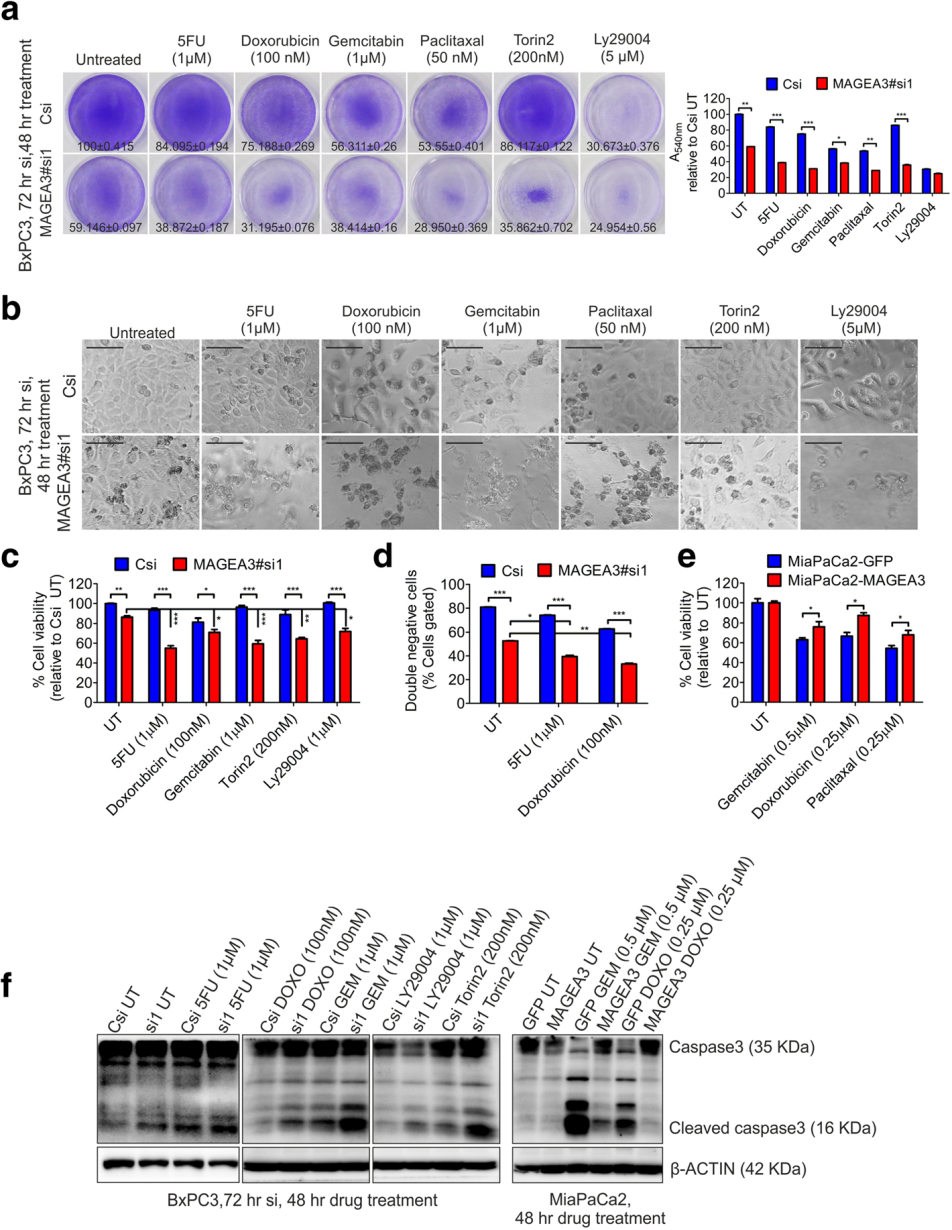
Targeting MAGEA3 increased the efficacy of cytotoxic/genotoxic agents in vitro*.* **a** Cystal violet stained images of the plates and the corresponding quantification bar graph for indicated conditions showing reduced viability of MAGEA3 depleted cells. The values represent percentage viability calculated using the formulae A_540nm_ (treated)/A_540nm_ (Untreated, Csi) X 100, Mean ± SEM, *n* = 3. * = *p* < 0.05, ** = *p* < 0.005 and *** = *p* < 0.001, *n* = 3. **b** Bright field micrographs (scale bar = 100 μm) showing reduced number of healthy cells (intact cell membrane and attached to culture plate) in MAGEA3 depleted condition in response to indicated compounds. **c**, **d** Cell viability assay (MTT assay graph; c and FACS analysis, showing both annexinV negative and propidium iodide negative cell population; **d**) shows decreased viable cells in MAGEA3 depleted condition in response to indicated molecules/agents (after 72 h siRNA and after 48 h drug treatment). The values presented in graph are Mean ± SEM, *n* = 3. * = *p* < 0.05, ** = *p* < 0.005, *** = *p* < 0.001. **e** MTT assay showing ectopic expression of MAGEA3 gives survival advantage to cancer cells in presence of various cytotoxic drugs (48 h drug treatment). The values presented in graph are Mean ± SEM, *n* = 3. * = *p* < 0.05. **f** Immunoblot showing increased apoptotic cell marker cleaved caspase3 upon MAGEA3 depletion in BxPC3 cells and the same is decreased upon ectopic expression of MAGEA3 in MiaPaCa2 cells at indicated conditions. 5FU, 5-Fluorouracil; DOXO, Doxorubicin, GEM, Gemcitabine

In our present study, we observed MAGEA3 as a survival factor for the cancer cells during GF-deprived stress condition. Further, we were interested in evaluating the contribution of MAGEA3 towards cancer cells survival during genotoxic and metabolic stress by using existing therapeutic molecules and metabolic switch modulators. Our results showed that MAGEA3 also has a significant role in providing a survival advantage to the cancer cells during the above-mentioned stress condition (Fig. 7).

## Discussion

In addition to the existing list of oncogenes/proto-oncogenes, recent reports on the identification of cancer-specific genes and their potential in cancer pathogenesis lead to the identification of new molecular targets [1–4, 11]. For a long time, MAGEA3 has been on light for hope as cancer immunotherapeutic [63, 64]; however, recent findings emphasizes on its functional role in the pathogenesis of different malignancies [16, 20, 22, 27]. In the current study, we report the cancer-promoting/favoring role of MAGEA3 in PCA. Our findings suggest that the PCCs expressing MAGEA3 are dependent on it to survive and thrive both in growth factor enriched and growth factor depleted condition (Figs. 1 and 2). However the cells, those are negative for MAGEA3 expression, don’t depend on it when desired growth factors are available (Fig. 1), but in growth factor limiting condition, ectopic expression of MAGEA3 is able to help the cancer cells to survive (Fig. 2 and Additional file 4: Figure S3). The cell lines used in this study have different oncogenic KRAS and P53 status (BxPC3 has WT-KRAS and Mut-TP53; AsPC1, MiaPca2 and PANC1 have Mut-KRAS and Mut-TP53) [65]; however, in the current study, we have not experimentally explored the correlation between KRAS and P53 status with MAGEA3 function. Hence, future studies in these aspects might elucidate the influence of common genetic aberrations on MAGEA3 function in pancreatic cancer cells.

In cancer cells, impaired autophagy was reported to be an important protumorigenic mechanism that supports the metabolic switching of cancer cells and favors Warburg’s effect [15, 16, 20, 56, 57]. Though early autophagy inhibition during GF-deprivation may provide pro-survival gain; prolonged autophagy inhibition can be lethal during continuous energy stress [56, 57]. Our study demonstrates that MAGEA3 reduces the autophagy level in PCCs, which agrees with earlier reports [15–17, 20]. Importantly, the reduced autophagy level during growth factor limitation provided a pro-survival advantage to the MAGEA3 over-expressing PCCs. Cancer cells have been reported to produce and use important chemokines in survival signalling pathways, which can be a potential factor responsible for the survival of cancer cells in prolonged GF-deprivation [58, 59, 66, 67]. Upon ectopic expression of MAGEA3, CCL2 and survivin were found to be up-regulated; conversely, upon MAGEA3 depletion both CCL2 and survivin level was reduced. A previous report had already shown the integrated role of CCL2 and survivin in reducing autophagy and providing survival advantage [60, 61]. Acquisition of intrinsic properties to overcome various metabolic stresses including GF-deprivation is one of the essential hallmarks of cancer cells [68, 69]. In cell line based in vivo tumor models, although the cells that are used to generate tumors are already transformed, but in absence of proper angiogenesis (during the initial establishment of tumors and at the core region of the fast-growing tumors) many cancer cells encounter sustained GF-deprivation and succumb to death. In our study, the presence of more number of cancer cells in MAGEA3 overexpressing tumors might be due to better survival and/or proliferation of cancer cells in tumor tissues (Fig. 6d and e). Our in vitro studies have provided convincing evidence that shows the role of MAGEA3-mediated autophagy inhibition as a potential mechanism through which MAGEA3 directly promotes survival of GF-deprived PCCs in vitro. However, in the in vivo context, in addition to this direct effect, MAGEA3 might have also indirectly affected the overall tumor growth by modulating different stromal events like angiogenesis. CCL2 is known to promote angiogenesis in different cancers [70–72]; hence, MAGEA3-mediated CCL2 overexpression in pancreatic cancer cells (Fig. 4c-e and g) might have also indirectly contributed to the overall tumor growth in vivo*,* which warrants further investigation. We also found that MAGEA3 is able to upregulate survivin in mouse primary pancreatic epithelial cells (Fig. 4i), which indicates the possible oncogenic role of MAGEA3 and needs further investigation. Our study involves the molecule MAGEA3 which is highly similar to MAGEA6. Although MAGEA3 and MAGEA6 are two different gene products, but due to their high similarity at protein level (> 90%), in different literatures both the genes have been reported together (MAGEA3/6) [16]. In the current study, we have used MAGEA3 coding sequence for its overexpression; however, the siRNA used by us might act on both the genes. Hence, we believe that the findings of the current study might also be true for MAGEA6 function in PCA.

Together, our study confirms the survival advantage provided by MAGEA3 during the hostile condition to PCCs. Moreover, the results of this study provide a rationale to target MAGEA3 and/or associated molecules like CCL2 for personalized PCA therapy. Further, we targeted MAGEA3 in PCCs and observed enhanced cytotoxic effect of various existing molecules upon MAGEA3 depletion but the cytotoxic effect is reduced when MAGEA3 is overexpressed in PCCs. Thus, it cautious the strategy to overexpress molecules like MAGEA3 in pancreatic cancer cells, which will make them more immunogenic like in other cancers [73–76] but may have a serious negative consequence. In the future, along with existing chemotherapy, MAGEA3-targeted therapy can be explored for a better therapeutic approach against PCA.

## Conclusion

Our study provides experimental evidence that suggests MAGEA3 is an important survival molecule for pancreatic cancer cells under metabolic and genotoxic stress conditions. The mechanistic study revealed that CCL2 and/or survivin are two possible functional mediators of MAGEA3. Thus, we propose that targeting MAGEA3 may have a better impact on PCA therapy.

## Additional files

Additional file 1: **Table S1.** (Primers). **Table S2.** (siRNA sequences). **Table S3.** (Antibodies). **Table S4.** (Reagents). **Table S5.** (Constructs). **Table S6.** (Softwares). (DOC 143 kb)

Additional file 2: **Figure S1.** Isolation and characterisation of mouse pancreatic epithelial cells. a Bright field micrograph of mouse pancreatic epithelial cells along with fibroblast cells on collagen coated culture plate after 24 h of isolation and seeding. Scale bar = 200 μm. b Bright field micrograph showing the reduced fibroblast cells (differentiated completely and stopped dividing) and attached epithelial cells on collagen coated culture plate after 5 days of 1st split. Scale bar =200 μm. c Bright field micrograph showing homogeneous population of mouse pancreatic epithelial cells. Scale bar =200 μm. d Immunofluorescence staining confirms the isolated cells are of epithelial origin. E-cadherin is used as epithelial marker. Images captured with 40X objective, scale bar = 20 μm. e Bright field micrograph showing no gross change in morphology after overexpressing the mouseKRAS^G12D^ or MAGEA3 or MAGEA3-HA or GFP proteins in the isolated mouse pancreatic epithelial cells (stable cells, selected with puromycin at a concentration of 3 μg/mL). Scale bar = 200 μm. f Immunoblot showing overexpression of mouseKRAS^G12D^ or huMAGEA3 or huMAGEA3-HA in mouse primary pancreatic epithelial stable cells. (JPG 2462 kb)

Additional file 3: **Figure S2.** MAGEA3 is expressed in pancreatic cancer cells. a, b qPCR (a) and immunoblot (b) analysis showing the differential expression of MAGEA3 in different pancreatic cancer cell lines. dCT = CT_MAGEA3_ – CT_18S_. Protein lysates from LNCap prostate cancer cells and PANC-1 cells transfected with *MAGEA3* overexpression construct (PANC1-MAGEA3) are used as positive control for MAGEA3 expression; β-actin is used as loading control. c-e Immunoblotting showing the indicated stable cell subjected to different doses of doxycycline (c, * above the band indicates leaky expression) and duration of treatment (d) to induce the MAGEA3-HA protein or native MAGEA3 protein (e, * above the band indicates leaky expression) in *tet-on* regulated system. f qPCR analysis of the indicated cells showing the basal level of *MAGEA3* expression in generated AsPC1-MAGEA3 stable cells without doxycycline induction and the level is further increased upon doxycycline treatment (100 ng/mL) for 24 h. The fold change is calculated using the formulae 2^^-ΔΔCT^. g Immunoblot analysis showing that the HA-tagged MAGEA3 is detected with the same anti-MAGEA3 antibody that detects native MAGEA3 in the indicated stable cells (constitutive expression system). h, i BxPC3 cells are transfected with indicated siRNA for 72 h and the level of *MAGEA3* is quantified by qPCR assay, bar graph; *18S rRNA* is used as loading control (h) or western blot, β-actin used as loading control (i). *** = *p* < 0.001, *n* = 3. (JPG 1125 kb)

Additional file 4: **Figure S3.** Effect of MAGEA3 overexpression on PCCs survival. a Crystal violet stained plate images and the adjacent bar graph (corresponding quantification, Mean ± SEM, *n* = 3) showing survival advantage conferred by MAGEA3 in MiaPaCa2 cells upon ectopic expression of MAGEA3 by regulated expression system (TRE-MAGEA3) and constitutive expression system (MAGEA3 or MAGEA3-FLAG) in growth factor deprived condition (0% FBS). * = *p* < 0.05 and ** = *p* < 0.005, *n* = 3. b Cell viability assay (MTT assay) showing more viable cells upon MAGEA3 expression in compared to parental cells. *** = *p* < 0.001, *n* = 3. c Immunoblot analysis showing reduced autophagic marker and increased survivin level in MAGEA3 expressing MiaPaCa2 cells compared to control cells in growth factor deprived condition. d Cell viability assay (MTT assay) showing more viable cells upon MAGEA3 expression in compared to parental cells. *** = *p* < 0.001, *n* = 3. e, f Immunoluorescence staining (e, scale bar 25 μm, 63X objective) and immunoblot (f) showing reduced level of autophagic marker LC3-II in PANC1 cells expressing MAGEA3 in compared to control cells. (JPG 2137 kb)

Additional file 5: **Figure S4**. MAGEA3 provides survival advantages to the pancreatic cancer cells in spheroid culture model. A Micrographs of spheroid (scale bar = 500 μm) showing bigger spheroid in case of MAGEA3 ectopic expression. b Cell viability assay by trypan blue dye exclusion cell counting method showing the percentage of trypan blue negative cells per spheroid at indicated conditions. c Immunoblot of proteins isolated from spheroids showing the expression of MAGEA3 or MAGEA3-HA, * above the band indicates leaky protein expression. d, e Immunohistochemical staining of spheroids for Ki67 (proliferation marker), scale bar = 200 μm, 10X objective; magnified images with yellow box inside, scale bar = 50 μm, 40X objective; image showing yellow arrow head (counted as negative for Ki67) and red arrow head (counted as positive for Ki67), scale bar = 25 μm (d). Bar graph showing quantification of % positive Ki67 cancer cells in spheroid. *** = *p* < 0.001, *n* = 4 (no. of fields counted per spheroid) (e). (JPG 1835 kb)

Additional file 6: **Figure S5.** Viability of MAGEA3 expressing cells in response to auotphagy inducer or inhibitor. a-d MTT assay showing the overall viability of the cancer cells ectopically expressed with MAGEA3 in compared to control cells (a and b) and that of BxPC3 cells depleted with MAGEA3 (c and d) in response to autophagic conditions (0% FBS, 0% FBS+ Rapamycin, 0% FBS + BafilomycinA1, 0% FBS + Torin2, 0% FBS + Wortmanin). * = *p* < 0.05, ** = *p* < 0.005, *** = *p* < 0.001, *n* = 3. D+ represents doxycycline induction. e Immunoblot validating the reduced autophagic flux in MAGEA3 expressing cells compared to control cells. * above the band indicates the leaky expression of MAGEA3 or MAGEA3-HA protein. (JPG 1705 kb)

Additional file 7: **Figure S6.** Effect of MAGEA3 overexpression and downregulation in BxPC3 cells in vivo*.* a-c Digital images (a, scale bar 1 cm) of xenografts isolated from nude mice and graph (b, tumor volume and c, tumor weight) showing greater size and weight of tumors generated from BxPC3 cells overexpressing MAGEA3 constitutively. * = *p* < 0.05, *n* = 5. d-f Digital images (d, scale bar 1 cm) of tumors dissected from nude mice and graph (e, tumor volume and f, tumor weight) showing reduced tumor size and weight upon MAGEA3 knockdown. ** = *p* < 0.005, *n* = 3. g, h Immunoblot and PCR showing MAGEA3 level in the tumor samples. GFP, BxPC3-GFP cells; MAGEA3, BxPC3-MAGEA3 (constitutive expression system). sh-Ve, non targeting shRNA control and sh1, MAGEA3 targeting shRNA. i, j Immunohistochemical analysis of xenografts showing more number of BrdU and survivin positive cancer cells in case of MAGEA3 overexpressing BxPC3 cells in compared to control cells (BxPC3-GFP) and the number is reduced in case of MAGEA3 knocked down BxPC3 cells (BxPC3-sh1) in compared to control cells (BxPC3-sh-ve), Scale bar = 25 μm. The bar graph represents the quantification (j), the values plotted is Mean ± SEM. ** = *p* < 0.005, *n* = 3. (JPG 1683 kb)

## Data Availability

Data sharing not applicable to this article as no datasets were generated during the current study. The data generated or analyzed during this study are included in this article and its additional files.

## Abbreviations

BSA: Bovine serum albumin
BSL2: Biological safety levels
CT: Cancer testis
DAB: 3,3′-diaminobenzidine
DAPI: 4′,6-diamidino-2-phenylindole
DMEM: Dulbecco’s modified eagle’s medium
DMSO: Dimethyl sulfoxide
ECACC: European collection of authenticated cell cultures
ECL: Enhanced chemiluminescence
EGTA: Ethylene glycol-bis(β-aminoethyl ether)-N,N,N′,N′-tetraacetic acid
FBS: Foetal bovine serum
GF: Growth factor
HBSS: Hank’s balanced salt solution
MAGEA3: Melanoma associated antigen A3
Na_2_EDTA: Disodium ethylene diamine tetraacetic acid
NCBI: National center for biotechnology information
Neo^R^: Neomycin resistance
PBS: Phosphate-buffered saline
PCA: Pancreatic cancer
PCCs: Pancreatic cancer cells
PCR: Polymerase chain reaction
PVDF: Polyvinylidene difluoride
RPMI: Roswell park memorial institute medium
SDS: Sodium dodecyl sulfate
