# Supplementary material for: Functional and mechanistic studies reveal MAGEA3 as a pro-survival factor in pancreatic cancer cells
Source: J Exp Clin Cancer Res. 2019 Jul 8;38:294. doi: 10.1186/s13046-019-1272-2 (PMC6615156; doi:10.1186/s13046-019-1272-2)
Supplement: Supplementary file 1 — Table S1. (Primers). Table S2. (siRNA sequences). Table S3. (Antibodies). Table S4. (Reagents). Table S5. (Constructs). Table S6. (Softwares). (DOC 143 kb) [file 13046_2019_1272_MOESM1_ESM.doc]

**Additional File 1**

**Table S**1 (Primers)

| **Sl No** | **Primer Name** | **Primer sequence (5’ to 3’)** | **Used annealing temperature (°C)** | **Amplicon Size (bp)** |
| --- | --- | --- | --- | --- |
| 1 | **EcoRV**-huMAGEA3-F | GCG**GATATC**CATCATGCCTCTTGAGCAG | 60 | 967 bp (No stop codon)  973 bp (With stop codon) |
| 2 | **XhoI**- NST huMAGEA3-R | GCG**CTCGAG**CTCTTCCCCCTCTCTC | 60 |
| 3 | **XhoI**- ST huMAGEA3-R | GCG**CTCGAG**TCATCACTCTTCCCCCTCTC | 60 |
| 4 | **attB1**-MA3-F | **GGGGACAAGTTTGTACAAAAAAGCAGGCTT**CATGCCTCTTGAGCAGAG | 60 | attB1-MAGEA3-attB2 (1009 bp)  attB1-MAGEA3-HA-attB2 (1036 bp) |
| 5 | **attB2**-ST-MA3-R | **GGGGACCACTTTGTACAAGAAAGCTGGGTC**TCATCACTCTTCCCCCTCTC | 60 |
| 6 | **attB2**-ST-**HA** MA3-R | **GGGGACCACTTTGTACAAGAAAGCTGGGTCTCAGGCGTAGTCGGGCACGTCGTAGGGGTA**CTCTTCCCCCTCTCTC | 60 |
| 7 | **attB1-**muKRAS^G12D^-F | **GGGGACAAGTTTGTACAAAAAAGCAGGCTT**ATGACTGAGTATAAACTTGTG | 55 | attB1-muKRAS^G12D^-HA-attB2  (654bp) |
| 8 | **attB2-**ST- **HA** muKRAS^G12D^-R | **GGGGACCACTTTGTACAAGAAAGCTGGGTCTCAGGCGTAGTCGGGCACGTCGTAGGGGTA**CATAACTGTACACCTTGTCCTTGA |
| 9 | h MA3qPCR F | TCTTCAGCAAAGCTTCCAGTT | 55 | 192 |
| 10 | h MA3qPCR R | CGCCCTCTCTTGCGATTATG |
| 11 | H 18S F | GTAACCCGTTGAACCCCATT | 55 | 151 |
| 12 | H 18S R | CCATCCAATCGGTAGTAGCG |
| 13 | h Survivin F | TGACGACCCCATAGAGGAACA | 55 | 226 |
| 14 | h Survivin R | GAGGCCTCAATCCATGGCAG |

**Table S**2 (siRNA sequences)

| **Sl No** | **siRNA Name** | **siRNA sequence** | **Source** |
| --- | --- | --- | --- |
| 1 | MAGEA3#si1 | Sense 5'-3': GAUGGUUGAAUGAGCGUCAdTdT  Antisense 3'-5': dTdTCUACCAACUUACUCGCAGU | Eurogenetec |
| 2 | MAGEA3#si2 | Sense 5'-3': GGUAAAGAUCAGUGGAGGAdTdT  AntiSense 3'-5': dTdTCCAUUUCUAGUCACCUCCU | Eurogenetec |

Continued..

| **Sl No** | **siRNA Name** | **siRNA sequence** | **Source** |
| --- | --- | --- | --- |
| 3 | Control si | SR-CL000-005 - Control siRNA duplex negative control 5 nmol | Eurogenetec |
| 4 | Lenti-sh-MAGEA3si1 | Same sequence as that of MAGEA3#si1 | Sigma |
| 5 | Lenti-sh-Control |  | Sigma |

**Table S3** (Antibodies)

| **Sl No** | **Antibody** | **Dilution** | **Host** | **Cat No** | **Source** |
| --- | --- | --- | --- | --- | --- |
| 1 | Anti-MAGEA3 | 1:1000 | Mouse | WH0004102M1-100UG | Sigma |
| 2 | Anti-HA | 1:1000 (WB)  1:1600 (IF) | Rabbit | 3724 | Cell Signalling |
| 3 | Anti-Flag | 1:3000 | Mouse | F3165 | Sigma |
| 4 | Anti-β-actin | 1:3000 | Mouse | A1978 | Sigma |
| 5 | Anti-β-actin | 1:3000 | Rabbit | A2066 | Sigma |
| 6 | Anti-LC3-II | 1:1000 (WB)  1:300 (IF) | Rabbit | 3868 | Cell Signalling |
| 7 | Anti-CCL2 | 1:1000 | Mouse | NBP2-22115 | Novus |
| 8 | Anti-Survivin | 1:1000 (WB)  1:400 (IHC) | Rabbit | 2808 | Cell Signalling |
| 9 | Anti-ki67 | 1:100 IHC | Rabbit | VP-RM04 | Vector laboratories |
| 10 | Anti-E-Cadherin | 1:50 IF-IC | Mouse | 14472 | Cell Signalling |
| 11 | Anti-BrdU | 1:200 IHC | Mouse | 5292 | Cell Signalling |
| 12 | Anti-Mouse IgG (whole molecule)–Peroxidase Conjugate | 1:3000 (WB) | Goat | A4416 | Sigma |
| 13 | Anti-Rabbit IgG (whole molecule)–Peroxidase Conjugate | 1:3000 (WB) | Goat | A6154 | Sigma |
| 14 | Alexa-Flour-488-anti-Rabbit | 1:500 (IF-IC) | Goat | A11008 | Invitrogen |
| 15 | Alexa-Flour-594-anti-Rabbit | 1:500 (IF-IC) | Goat | A11037 | Invitrogen |
| 16 | Phalloidin-555 | 1:200 (IF-IC) |  | 8953 | Cell Signalling |
| 17 | Anti-CCl2 functional grade (Neutralization antibody) | 2ng/mL  CCL2 neutralization experiment | Armenian Hamster | 16-7096 | eBioscience |
| 18 | Armenian Hamster IgG isotype control | 2ng/mL  Control IgG for CCL2 neutralization experiment | Armenian Hamster | 14-4888 | eBioscience |

**Table S4** (Reagents)

| **Sl No** | **Reagent Name** | **Purpose** | **Cat No** | **Company** |
| --- | --- | --- | --- | --- |
| 1 | DMEM | Cell culture | P04-03590 | PAN biotech |
| 2 | RPMI | Cell culture | P04-16500 | PAN biotech |
| 3 | FBS | Cell culture | 10270-106 | Invitrogen |
| 4 | Penicillin & Streptomycin | Cell culture | 15140-122 | Invitrogen |
| 5 | Trypsin | Cell culture | 25200-056 | Invitrogen |
| 6 | RNAeasy mini kit | RNA isolation | 74104 | Qiagen |
| 7 | RNase-free-DNase set | Remove DNA from RNA | 79254 | Qiagen |
| 8 | High Capacity cDNA synthesis kit | First strand cDNA synthesis from RNA | 4368814 | Appield biosystems |
| 9 | MESA GREEN qPCR 2X MasterMix Plus 7.5 ml - 600 rxn (7,5 ml) | qPCR | RT-SY2X-03+WOU | Eurogentec |
| 10 | RNAiMax | siRNA transfection into cultured cells | 13778 | Invitrogen |
| 11 | Lipofectamine™ 3000 Transfection Reagent | plasmid transfection into cultured cells | L3000001 | Invitrogen |
| 12 | Pierce™ HA-Tag IP/Co-IP Kit | Pull down of HA tag protein | 26180 | Pierce |
| 13 | ProLong™ Gold Antifade Mountant with DAPI | Stain nucleus and mount | P36935 | Invitrogen |
| 14 | VECTASTAIN Elite ABC HRP Kit (Peroxidase, Universal), R.T.U. (Ready-to-Use) | IHC | PK-7200 | Vector Laboratories |
| 15 | DAB Peroxidase (HRP) Substrate Kit (with Nickel), 3,3’-diaminobenzidine | IHC | SK-4100 | Vector Laboratories |
| 16 | Mouse on Mouse (M.O.M.™) Blocking Reagent | IHC (BrdU) | MKB-2213 | Vector Laboratories |
| 17 | TOPO TA Cloning Kit  pCR 2.1-TOPO Vector | TA-cloning | 45-0641 | Invitrogen |
| 18 | Zero Blunt TOPO PCR Cloning Kit  pCR-Blunt II-TOPO Vector | Blunt end cloning | 45-0245 | Invitrogen |
| 19 | EcoRV | Restriction digestion enzyme | R0195S | NEB |
| 20 | XhoI | Restriction digestion enzyme | R0146S | NEB |
| 21 | T4 ligase | Ligation enzyme for cloning | M0202S | NEB |
| 22 | Gateway™ BP Clonase™ II Enzyme mix | Gateway cloning into donor vector | 11789020 | Invitrogen |
| 23 | Gateway™ LR Clonase™ II Enzyme mix | Gateway cloning into destination vector | 11791020 | Invitrogen |
| 24 | CalPhos™ Mammalian Transfection Kit | Transfection of lentiviral packaging and target plasmids into HEK293T cells | 631312 | Clontech |
| 25 | Lenti-X concentrator | Concentrate viral particles and increase viral titre | 631231 | Clontech |
| 26 | MTT | Cell viability assay | M2128-5G | Sigma |
| 27 | Doxycycline | Induce gene expression | D9891-1G | Sigma |
| 28 | WesternBright ECL kit | Chemiluminoscenct substrate for HRP | K-12045-D20 | Aadvansta |
| 29 | Western BLoTUltra Sensitive HRP Substrate | Chemiluminoscent substrate for HRP (femto level detection of protein) | T7104A | TaKaRa |
| 30 | Dead Cell Apoptosis Kit with Annexin V Alexa Fluor™ 488 &Propidium Iodide (PI) | Apoptosis Assay | V13241 | Invitrogen |

Continued..

| **Sl No** | **Reagent Name** | **Purpose** | **Cat No** | **Company** |
| --- | --- | --- | --- | --- |
| 31 | Antigen Unmasking Solution, Citric Acid Based | IHC | H-3300 | Vector Laboratories |
| 32 | Gemcitabine |  | G6423-10MG | Sigma |
| 33 | 5-Fluorouracil |  | F6627-5G | Sigma |
| 34 | Doxorubicin |  | 0215910105 - 5 mg | MP bio |
| 35 | Paclitaxal |  | T7402-5MG | Sigma |
| 36 | Ly29004 |  | L9908-1MG | Sigma |
| 37 | Torin2 |  | SML1224-5MG | Sigma |
| 38 | Wortmanin |  | W1628-1MG | Sigma |
| 39 | Rapamcin |  | R8781-200UL | Sigma |
| 40 | BafilomycinA1 |  | B1793-2UG | Sigma |
| 41 | Lenti-sh-MAGEA3si1 lentiviral particle | Stable cell generation | CSTVRS  08071811MN | Sigma |
| 42 | Lenti-sh-Control lentiviral particle | Stable cell generation | CSTVRS 08071811MN | Sigma |
| 43 | Human CCL2 Recombinant Protein |  | 14-8398 | eBioscience |
| 44 | Human CCL2 ELISA | CCL2 quantification | DCP00 | R&D Systems |

**Table S5** (Constructs)

| **Sl No** | **Construct Name** | **Use** | **Inserted gene** | **Tag** | **Selection Marker** | **Source** |
| --- | --- | --- | --- | --- | --- | --- |
| 1 | pCMV 3Tag 3A | Constitutive gene expression non viral vector | Empty | C-terminal 3X Flag tag | Kanamycin  Neomycin | Generous gift from Dr. S.K Mishra |
| 2 | pCMV-hMAGEA3 | Constitutive gene expression non viral construct | huMAGEA3 | No tag | Kanamycin  Neomycin | Generated during this study |
| 3 | pCMV-hMAGEA3-Flag | Constitutive gene expression non viral construct | huMAGEA3 | C-terminal 3X Flag tag | Kanamycin  Neomycin | Generated during this study |
| 4 | pLenti CMV Puro DEST (w118-1)  Adgene-17452 | Constitutive gene expression lentiviral destination vector | Empty | No tag | Ampicilin  Puromycin | Generous gift from Dr. Rajeeb K Swain |
| 5 | pLenti CMV GFP Puro (658-5)  Adgene-17448 | Constitutive gene expression lentiviral vector | GFP | No tag | Ampicilin  Puromycin | Generous gift from Dr. Rajeeb K Swain |
| 6 | pLenti CMV- hMAGEA3 | Constitutive gene expression lentiviral construct | huMAGEA3 | No tag | Ampicilin  Puromycin | Generated during this study |
| 7 | pLenti CMV- hMAGEA3-HA | Constitutive gene expression lentiviral construct | huMAGEA3-HA | C-terminal HA tag | Ampicilin  Puromycin | Generated during this study |

Continued..

| **Sl No** | **Construct Name** | **Use** | **Inserted gene** | **Tag** | **Selection Marker** | **Source** |
| --- | --- | --- | --- | --- | --- | --- |
| 8 | pLenti CMV- muKRAS^G12D^-HA | Constitutive gene expression lentiviral construct | muKRAS^G12D^-HA | C-terminal HA tag | Ampicilin  Puromycin | Generated during this study |
| 9 | pSIN-TRE | Tetracycline inducible gene expression lentiviral vector | Empty | C-terminal 3XHA tag | Ampicilin  Puromycin | Generous gift from Dr. Sunil K Raghav |
| 10 | pSIN-TRE- hMAGEA3 | Tetracycline inducible gene expression lentiviral construct | huMAGEA3 | No tag | Ampicilin  Puromycin | Generated during this study |
| 11 | pSIN-TRE- hMAGEA3-HA | Tetracycline inducible gene expression lentiviral construct | huMAGEA3-HA | C-terminal HA tag | Ampicilin  Puromycin | Generated during this study |
| 12 | pDONR221 | Donor vector |  |  | Kanamycin | Generous gift from Dr. Rajeeb K Swain |

**Table S**6 (Softwares)

| **Sl No** | **Software Name** | **Purpose** |
| --- | --- | --- |
| 1 | Microsoft Office 2007 | Manuscript drafting |
| 2 | Corel Draw X7 | Image Compiling |
| 3 | Graph Pad Prism 5 | Statistical Analysis |
| 4 | Endnote X6 | Reference Editing |
| 5 | FinchTV | Sanger Sequencing Data analysis |
| 6 | Snapgene Viewer | Vector Sequence and features analysis |
| 7 | ImageJ | Quantitative analysis of western blots and images |
| 8 | Image Lab V 6.0.0 build 25 | Western blot and agarose gel analysis |
| 9 | Flow Jo V10 | FACS data analysis |
