# Supplementary figures and images for: Functional and mechanistic studies reveal MAGEA3 as a pro-survival factor in pancreatic cancer cells
Source: J Exp Clin Cancer Res. 2019 Jul 8;38:294. doi: 10.1186/s13046-019-1272-2 (PMC6615156; doi:10.1186/s13046-019-1272-2)

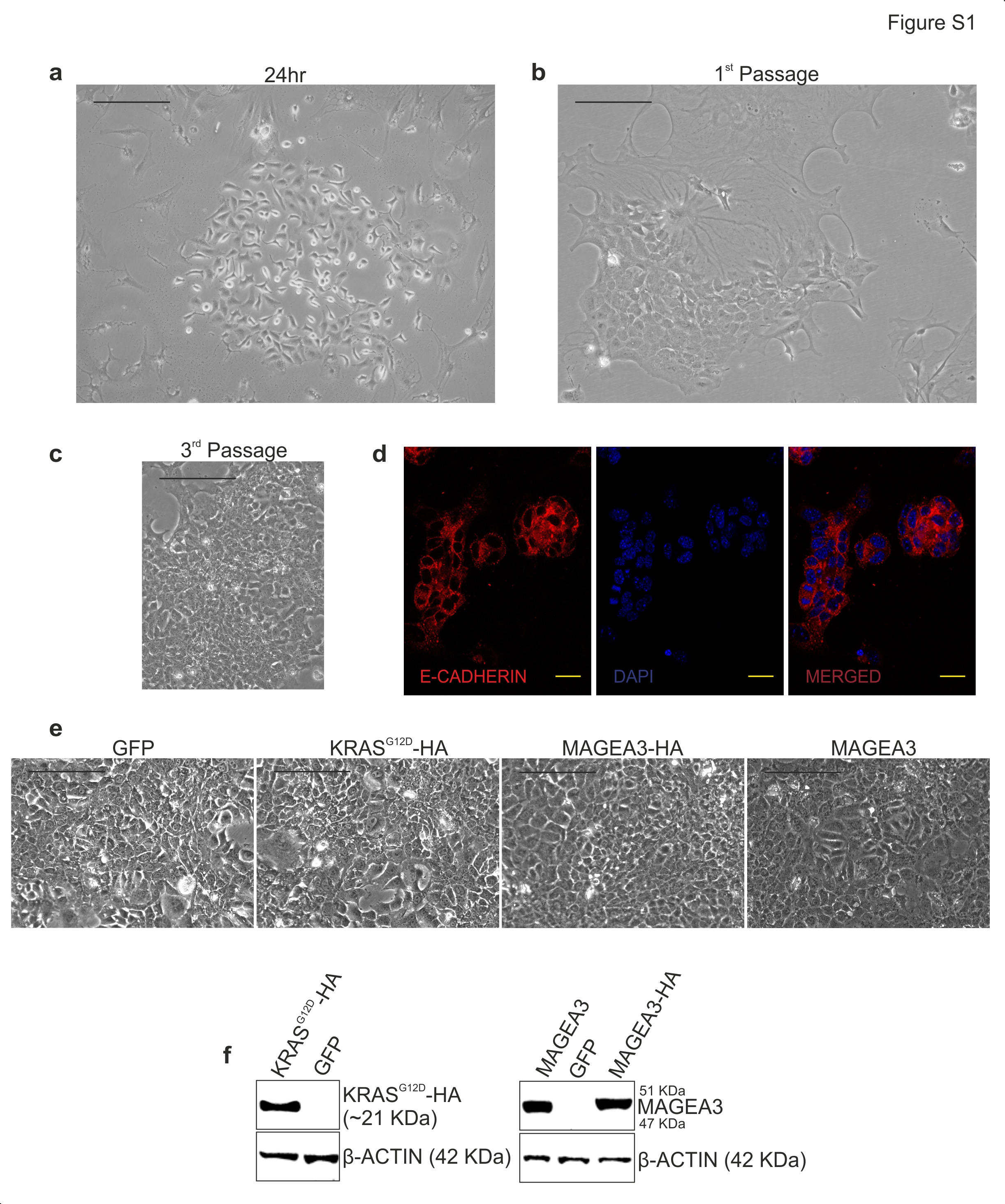

Supplement: Supplementary file 2 — Figure S1. Isolation and characterisation of mouse pancreatic epithelial cells. a Bright field micrograph of mouse pancreatic epithelial cells along with fibroblast cells on collagen coated culture plate after 24 h of isolation and seeding. Scale bar = 200 μm. b Bright field micrograph showing the reduced fibroblast cells (differentiated completely and stopped dividing) and attached epithelial cells on collagen coated culture plate after 5 days of 1st split. Scale bar =200 μm. c Bright field micrograph showing homogeneous population of mouse pancreatic epithelial cells. Scale bar =200 μm. d Immunofluorescence staining confirms the isolated cells are of epithelial origin. E-cadherin is used as epithelial marker. Images captured with 40X objective, scale bar = 20 μm. e Bright field micrograph showing no gross change in morphology after overexpressing the mouseKRASG12D or MAGEA3 or MAGEA3-HA or GFP proteins in the isolated mouse pancreatic epithelial cells (stable cells, selected with puromycin at a concentration of 3 μg/mL). Scale bar = 200 μm. f Immunoblot showing overexpression of mouseKRASG12D or huMAGEA3 or huMAGEA3-HA in mouse primary pancreatic epithelial stable cells. (JPG 2462 kb) [file 13046_2019_1272_MOESM2_ESM.jpg]

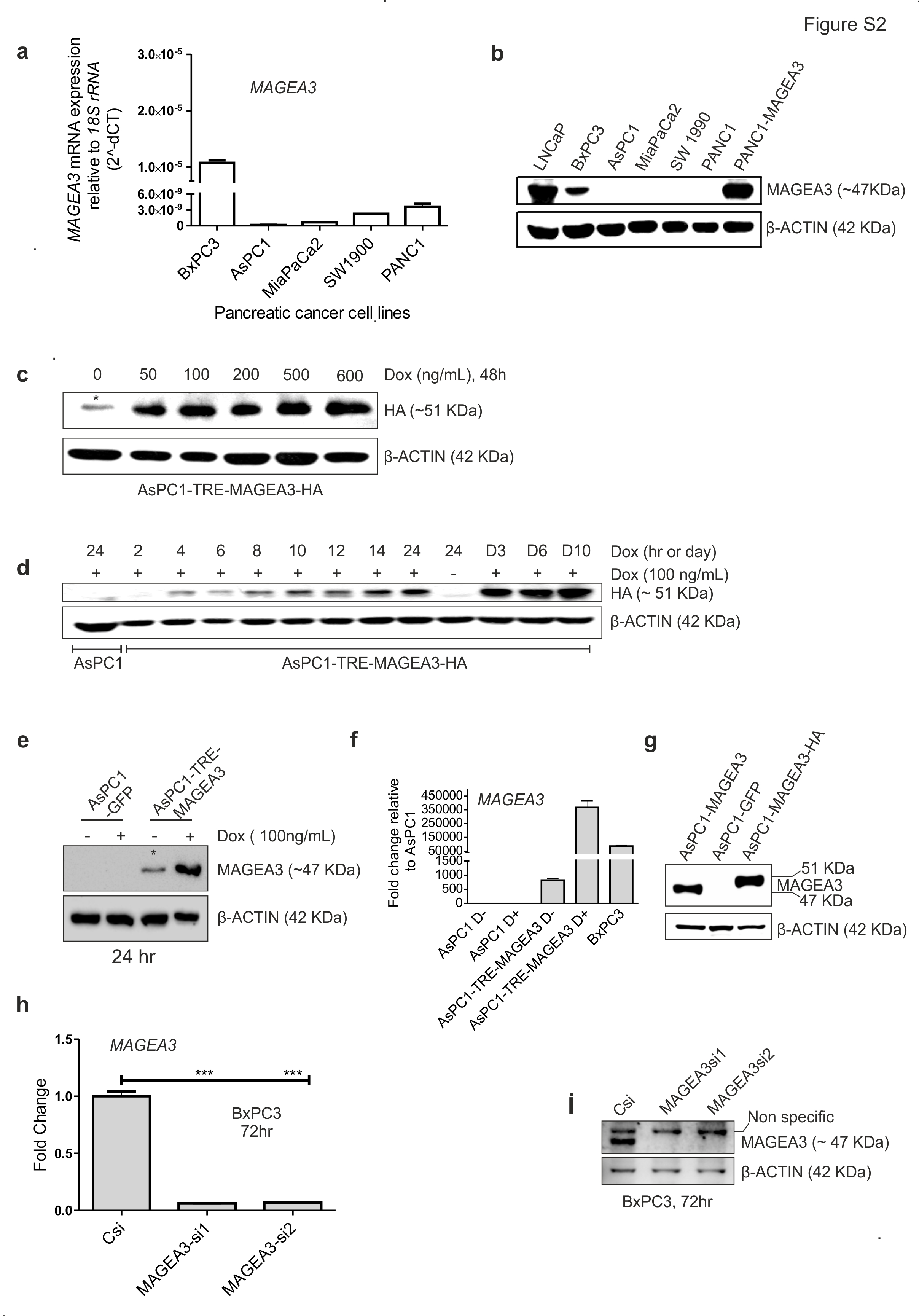

Supplement: Supplementary file 3 — Figure S2. MAGEA3 is expressed in pancreatic cancer cells. a, b qPCR (a) and immunoblot (b) analysis showing the differential expression of MAGEA3 in different pancreatic cancer cell lines. dCT = CTMAGEA3 – CT18S. Protein lysates from LNCap prostate cancer cells and PANC-1 cells transfected with MAGEA3 overexpression construct (PANC1-MAGEA3) are used as positive control for MAGEA3 expression; β-actin is used as loading control. c-e Immunoblotting showing the indicated stable cell subjected to different doses of doxycycline (c, * above the band indicates leaky expression) and duration of treatment (d) to induce the MAGEA3-HA protein or native MAGEA3 protein (e, * above the band indicates leaky expression) in tet-on regulated system. f qPCR analysis of the indicated cells showing the basal level of MAGEA3 expression in generated AsPC1-MAGEA3 stable cells without doxycycline induction and the level is further increased upon doxycycline treatment (100 ng/mL) for 24 h. The fold change is calculated using the formulae 2^-ΔΔCT. g Immunoblot analysis showing that the HA-tagged MAGEA3 is detected with the same anti-MAGEA3 antibody that detects native MAGEA3 in the indicated stable cells (constitutive expression system). h, i BxPC3 cells are transfected with indicated siRNA for 72 h and the level of MAGEA3 is quantified by qPCR assay, bar graph; 18S rRNA is used as loading control (h) or western blot, β-actin used as loading control (i). *** = p < 0.001, n = 3. (JPG 1125 kb) [file 13046_2019_1272_MOESM3_ESM.jpg]

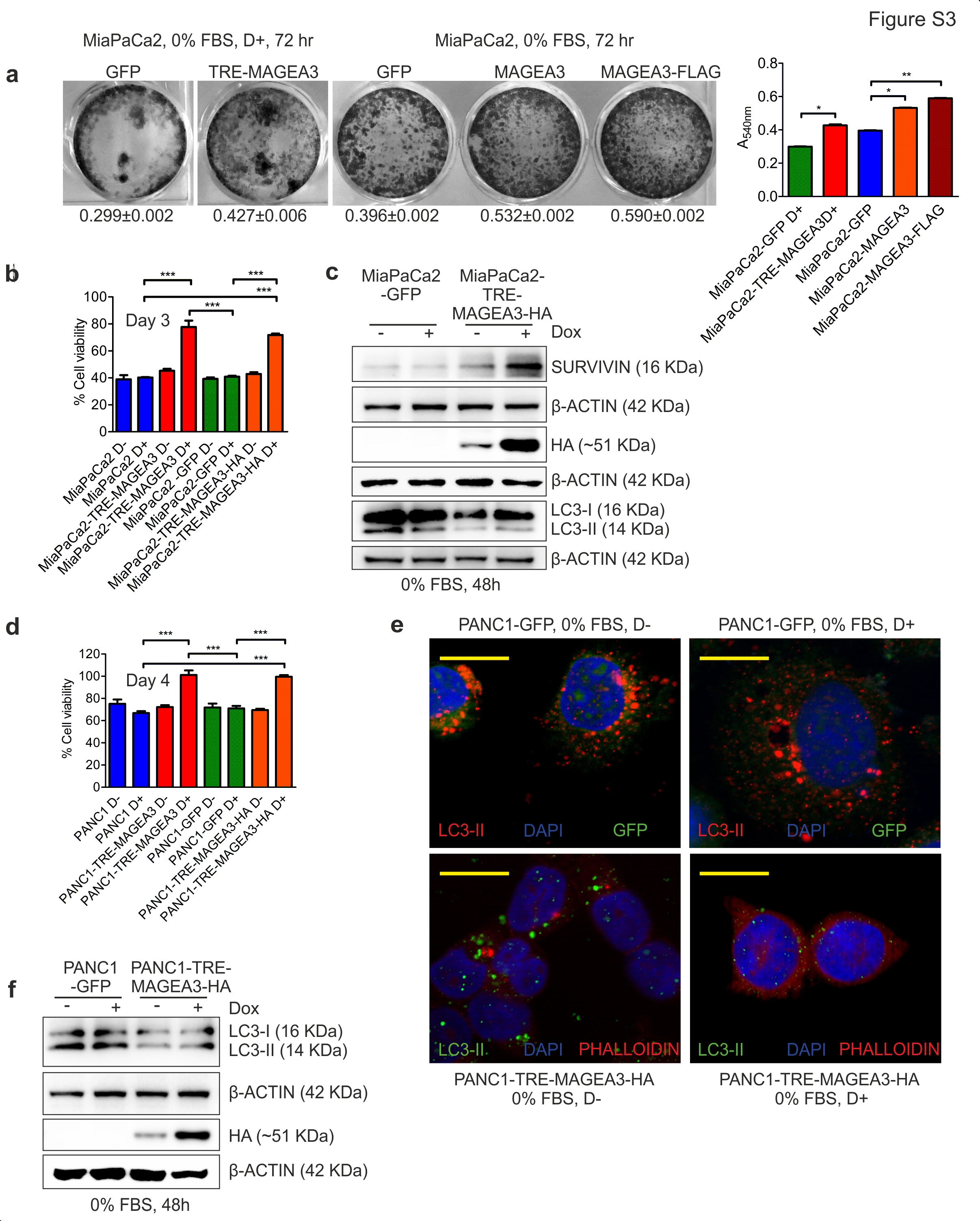

Supplement: Supplementary file 4 — Figure S3. Effect of MAGEA3 overexpression on PCCs survival. a Crystal violet stained plate images and the adjacent bar graph (corresponding quantification, Mean ± SEM, n = 3) showing survival advantage conferred by MAGEA3 in MiaPaCa2 cells upon ectopic expression of MAGEA3 by regulated expression system (TRE-MAGEA3) and constitutive expression system (MAGEA3 or MAGEA3-FLAG) in growth factor deprived condition (0% FBS). * = p < 0.05 and ** = p < 0.005, n = 3. b Cell viability assay (MTT assay) showing more viable cells upon MAGEA3 expression in compared to parental cells. *** = p < 0.001, n = 3. c Immunoblot analysis showing reduced autophagic marker and increased survivin level in MAGEA3 expressing MiaPaCa2 cells compared to control cells in growth factor deprived condition. d Cell viability assay (MTT assay) showing more viable cells upon MAGEA3 expression in compared to parental cells. *** = p < 0.001, n = 3. e, f Immunoluorescence staining (e, scale bar 25 μm, 63X objective) and immunoblot (f) showing reduced level of autophagic marker LC3-II in PANC1 cells expressing MAGEA3 in compared to control cells. (JPG 2137 kb) [file 13046_2019_1272_MOESM4_ESM.jpg]

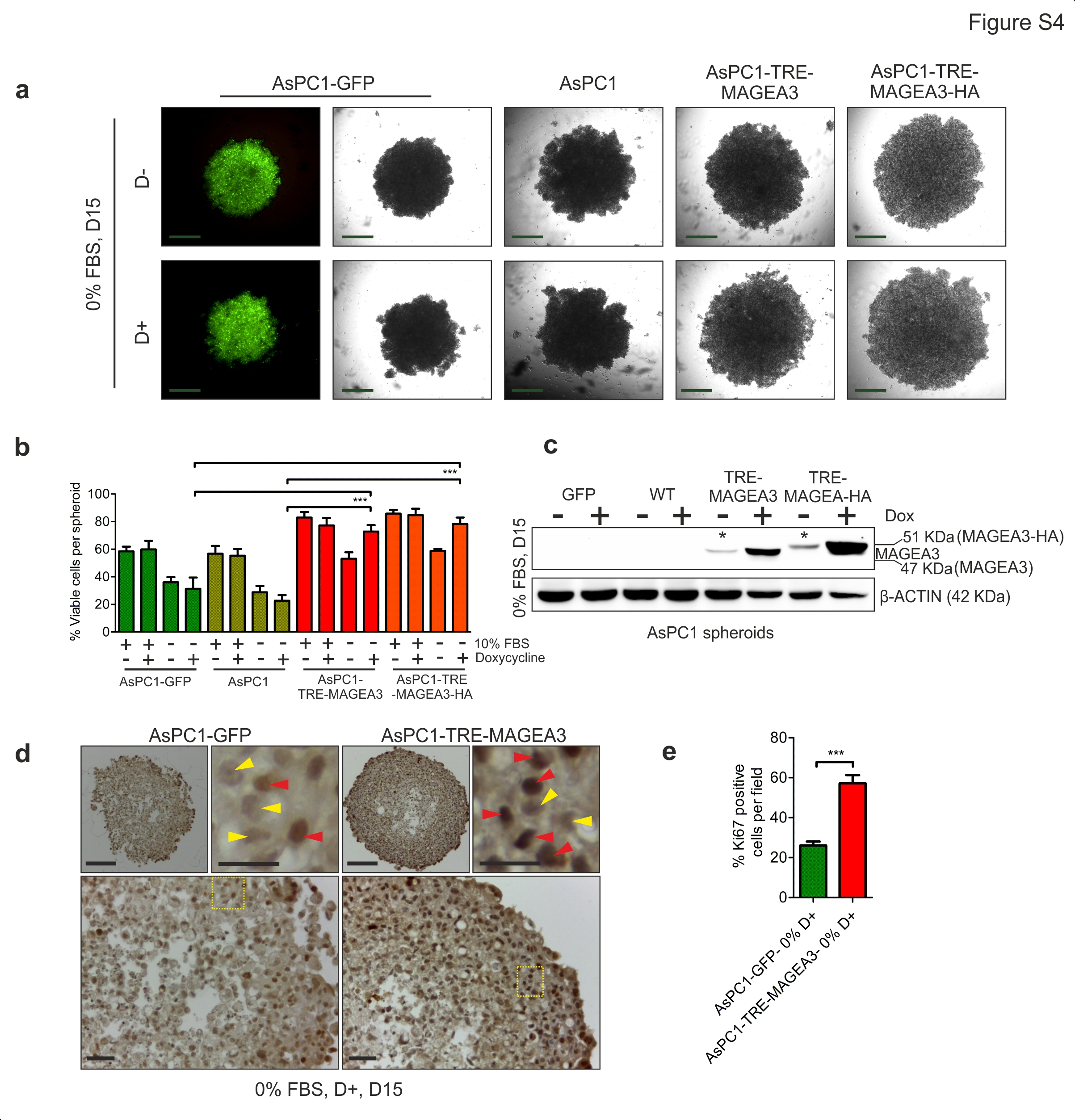

Supplement: Supplementary file 5 — Figure S4. MAGEA3 provides survival advantages to the pancreatic cancer cells in spheroid culture model. A Micrographs of spheroid (scale bar = 500 μm) showing bigger spheroid in case of MAGEA3 ectopic expression. b Cell viability assay by trypan blue dye exclusion cell counting method showing the percentage of trypan blue negative cells per spheroid at indicated conditions. c Immunoblot of proteins isolated from spheroids showing the expression of MAGEA3 or MAGEA3-HA, * above the band indicates leaky protein expression. d, e Immunohistochemical staining of spheroids for Ki67 (proliferation marker), scale bar = 200 μm, 10X objective; magnified images with yellow box inside, scale bar = 50 μm, 40X objective; image showing yellow arrow head (counted as negative for Ki67) and red arrow head (counted as positive for Ki67), scale bar = 25 μm (d). Bar graph showing quantification of % positive Ki67 cancer cells in spheroid. *** = p < 0.001, n = 4 (no. of fields counted per spheroid) (e). (JPG 1835 kb) [file 13046_2019_1272_MOESM5_ESM.jpg]

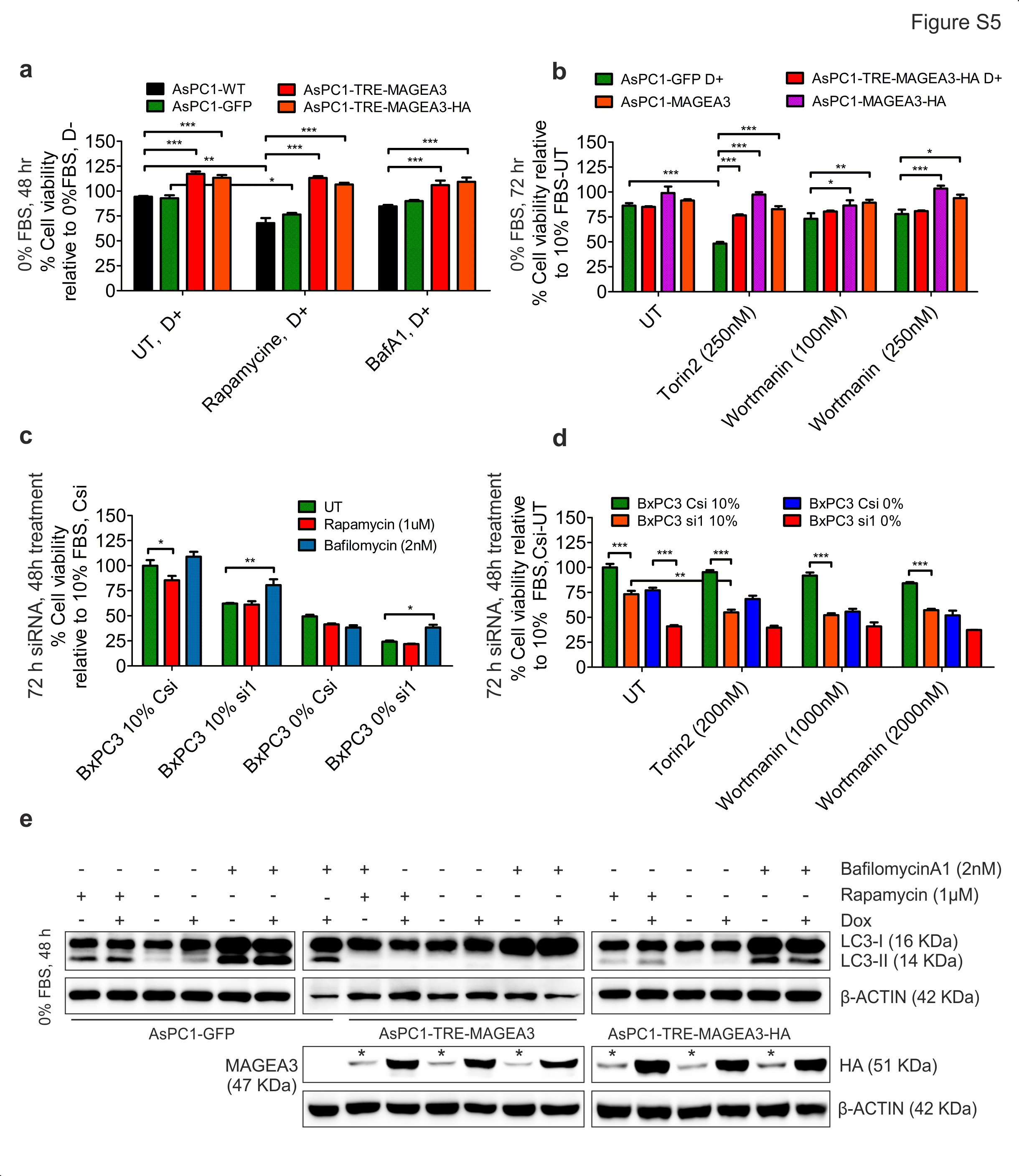

Supplement: Supplementary file 6 — Figure S5. Viability of MAGEA3 expressing cells in response to auotphagy inducer or inhibitor. a-d MTT assay showing the overall viability of the cancer cells ectopically expressed with MAGEA3 in compared to control cells (a and b) and that of BxPC3 cells depleted with MAGEA3 (c and d) in response to autophagic conditions (0% FBS, 0% FBS+ Rapamycin, 0% FBS + BafilomycinA1, 0% FBS + Torin2, 0% FBS + Wortmanin). * = p < 0.05, ** = p < 0.005, *** = p < 0.001, n = 3. D+ represents doxycycline induction. e Immunoblot validating the reduced autophagic flux in MAGEA3 expressing cells compared to control cells. * above the band indicates the leaky expression of MAGEA3 or MAGEA3-HA protein. (JPG 1705 kb) [file 13046_2019_1272_MOESM6_ESM.jpg]

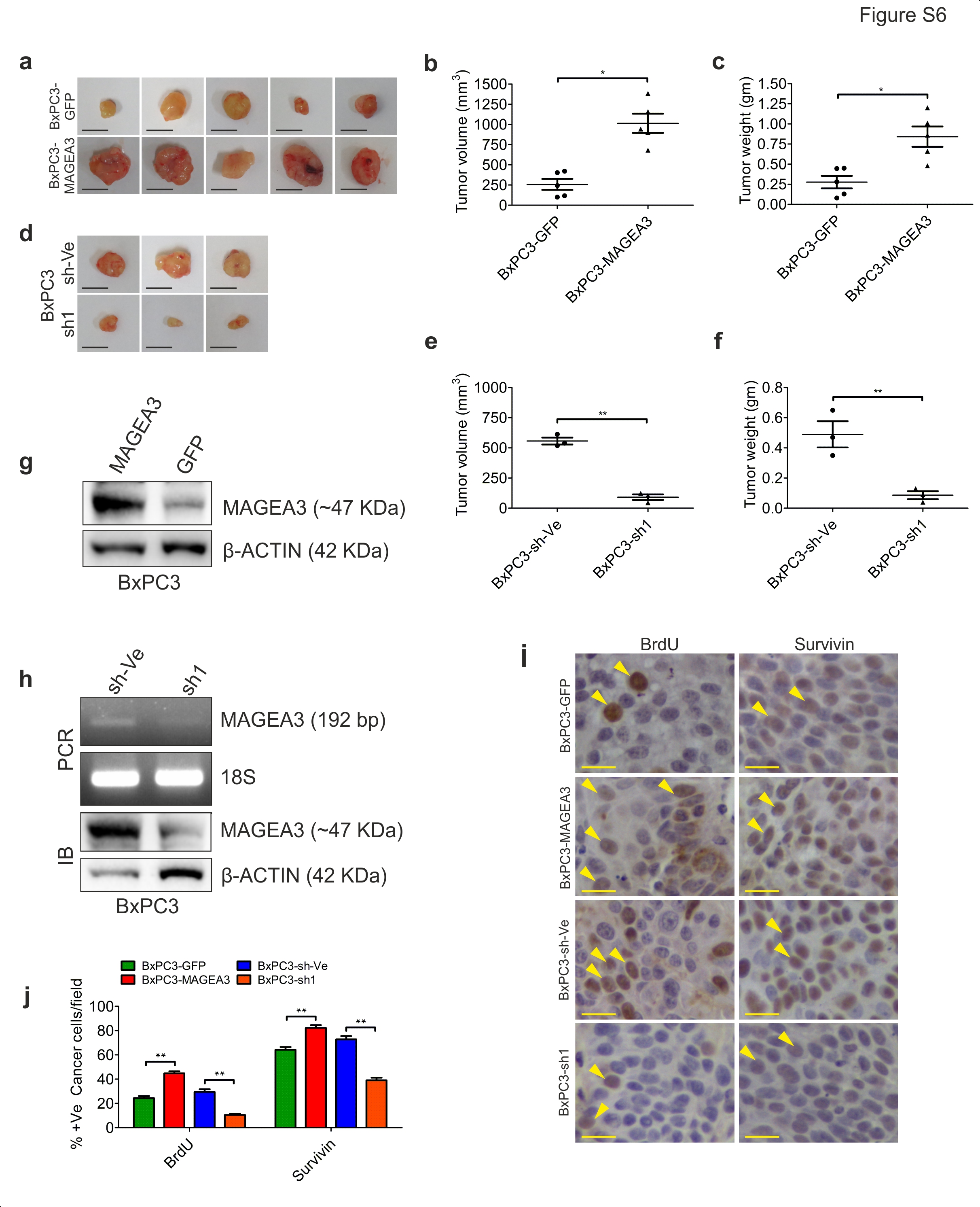

Supplement: Supplementary file 7 — Figure S6. Effect of MAGEA3 overexpression and downregulation in BxPC3 cells in vivo. a-c Digital images (a, scale bar 1 cm) of xenografts isolated from nude mice and graph (b, tumor volume and c, tumor weight) showing greater size and weight of tumors generated from BxPC3 cells overexpressing MAGEA3 constitutively. * = p < 0.05, n = 5. d-f Digital images (d, scale bar 1 cm) of tumors dissected from nude mice and graph (e, tumor volume and f, tumor weight) showing reduced tumor size and weight upon MAGEA3 knockdown. ** = p < 0.005, n = 3. g, h Immunoblot and PCR showing MAGEA3 level in the tumor samples. GFP, BxPC3-GFP cells; MAGEA3, BxPC3-MAGEA3 (constitutive expression system). sh-Ve, non targeting shRNA control and sh1, MAGEA3 targeting shRNA. i, j Immunohistochemical analysis of xenografts showing more number of BrdU and survivin positive cancer cells in case of MAGEA3 overexpressing BxPC3 cells in compared to control cells (BxPC3-GFP) and the number is reduced in case of MAGEA3 knocked down BxPC3 cells (BxPC3-sh1) in compared to control cells (BxPC3-sh-ve), Scale bar = 25 μm. The bar graph represents the quantification (j), the values plotted is Mean ± SEM. ** = p < 0.005, n = 3. (JPG 1683 kb) [file 13046_2019_1272_MOESM7_ESM.jpg]
